# Open access tools for quality-assured and efficient data entry in a large, state-wide tobacco survey in India

**DOI:** 10.1080/16549716.2017.1394763

**Published:** 2017-11-02

**Authors:** Hemant Deepak Shewade, E Vidhubala, Divyaraj Prabhakar Subramani, Pranay Lal, Neelam Bhatt, C. Sundaramoorthi, Rana J. Singh, Ajay M. V. Kumar

**Affiliations:** ^a^ Department of Operational Research, International Union Against Tuberculosis and Lung Disease (The Union), South-East Asia Office, New Delhi, India; ^b^ Department of Psycho-oncology, Cancer Institute (Women’s India Association), Chennai, India; ^c^ Department of Tobacco control and noncommunicable diseases, International Union Against Tuberculosis and Lung Disease (The Union) South-East Asia Office, New Delhi, India; ^d^ Center for Operational Research, International Union Against Tuberculosis and Lung Disease (The Union), Paris, France

**Keywords:** Tobacco use prevalence, tobacco surveillance, data entry, EpiData, data documentation, Global Adult Tobacco Survey, GATS, open access tools, Tamil Nadu, India

## Abstract

**Background**: A large state-wide tobacco survey was conducted using modified version of pretested, globally validated Global Adult Tobacco Survey (GATS) questionnaire in 2015–22016 in Tamil Nadu, India. Due to resource constrains, data collection was carrid out using paper-based questionnaires (unlike the GATS-India, 2009–2010, which used hand-held computer devices) while data entry was done using open access tools. The objective of this paper is to describe the process of data entry and assess its quality assurance and efficiency.

**Methods**: In EpiData language, a variable is referred to as ‘field’ and a questionnaire (set of fields) as ‘record’. EpiData software was used for double data entry with adequate checks followed by validation. Teamviewer was used for remote training and trouble shooting. The EpiData databases (one each for each district and each zone in Chennai city) were housed in shared Dropbox folders, which enabled secure sharing of files and automatic back-up. Each database for a district/zone had separate file for data entry of household level and individual level questionnaire.

**Results**: Of 32,945 households, there were 111,363 individuals aged ≥15 years. The average proportion of records with data entry errors for a district/zone in household level and individual level file was 4% and 24%, respectively. These are the errors that would have gone unnoticed if single entry was used. **T**he median (inter-quartile range) time taken for double data entry for a single household level and individual level questionnaire was 30 (24, 40) s and 86 (64, 126) s, respectively.

**Conclusion**: Efficient and quality-assured near-real-time data entry in a large sub-national tobacco survey was performed using innovative, resource-efficient use of open access tools.

## Background

The Global Adult Tobacco Survey (GATS)-India (2009–2010) was conducted adopting a standard methodology in 29 states and two union territories of India (*n* = 69,296) which provided regional (north, central, west, south, east, and north-east) and national-level information on key tobacco control indicators. Hand-held computers, used by the data collectors, combined the processes of data collection and data entry into one which was facilitated by complex skip patterns in the questionnaire as well as built-in validity checks for quality control [1].

Despite the methodological rigour, scope for improvement remained. GATS-India (2009–2010) did not provide precise state-level estimates due to inadequate sample size largely due to errors in base population estimates [2]. As a result, some state-level and district-level estimates were not available for local policymakers. In addition, there were inconsistencies in the data. Statewise data after removal of missing variables did not have sufficient sample size and were not representative. There could be two reasons for this. First, the survey was conducted in 19 other languages and it was not clear whether the survey instrument was pre-tested in all languages. Secondly, the pilot was conducted well over a year before data collection commenced [2].

Keeping this in mind, a state-wide tobacco survey, Tamil Nadu Tobacco Survey (TNTS), was conducted in 2015, with a district-wise focus, in the state of Tamil Nadu, south India. The implementing agency of TNTS, Cancer Institute (Women’s India Association), Chennai, India did not have funding support for hand-held computers and therefore used traditional, paper-based survey questionnaires to collect data and data entry had to be planned separately. Technical support for data entry was provided by The International Union Against Tuberculosis and Lung Disease (The Union), South-East Asia Office, New Delhi, India.

Double data entry and validation is considered the gold standard for reducing data entry errors (quality assurance). In this, data are entered independently twice and the two databases are compared for discordances, followed by their resolution by referring to the original data collection forms [3,4]. To achieve this, we replicated a model of combining open-access tools for quality-assured data entry (previously described in an operational research setting) in a large sub-national tobacco survey (TNTS) in a resource-constrained setting [5]. If this model of data entry proves to be quality-assured and efficient, then it has the potential to be replicated in other settings.

Therefore, the *objective* of this process paper is two-fold: first, to describe the data entry process which combined the use of open access tools like EpiData (with focus on checks for data entry and double data entry process), Dropbox and TeamViewer; secondly, to describe indicators pertaining to quality assurance (data entry errors) and efficiency of data entry (average time taken to double enter one questionnaire). The actual findings of the TNTS will be published elsewhere.

## Methods

### Setting

**Figure 1.**
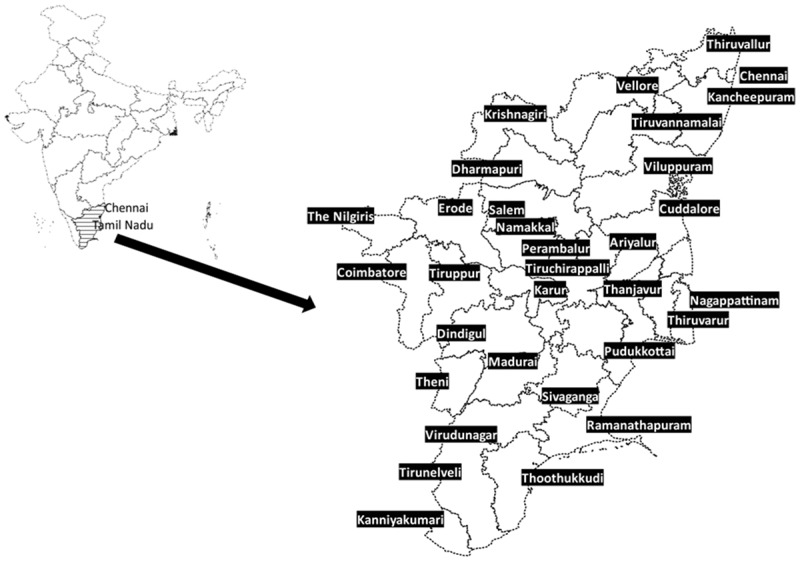
Map of India depicting the state of Tamil Nadu (India) with the capital city of Chennai and thirty one districts.

Tamil Nadu, a state in south India, has 31 districts with Chennai as the capital city (Figure 1). It has a population of 72 million (rural:urban ratio 1:1) [6].

### Data collection

Under TNTS, each of the 31 districts was divided into urban and rural areas except Chennai city which was divided into 15 zones, each zone further divided into slum and non-slum areas. Data were collected from all the 31 districts of the state and 15 zones of Chennai city between March and November, 2015. Estimated sample size of 100,000 people was divided among urban and rural areas of districts and slum and non-slum areas of zones in Chennai city using Probability Proportional to Size sampling [6]. Primary and secondary sampling units in rural areas were households and villages, respectively. Primary, secondary and tertiary sampling units in urban areas were households, census enumeration blocks and wards, respectively. All individuals (≥15 years), males and females, in the selected household were interviewed.

Data collection was carried out using a modified version of the GATS questionnaire [7]. The questionnaire was divided into two parts: household level and individual level questionnaire. Each household and individual was provided with a unique identifier. Details of the methodology will be published elsewhere.

### Electronic data entry

A responsible person from The Union (HDS) coordinated the process with a responsible offficial from The Cancer Institute (WIA) (DPS).

#### Open access tools

We used the following three open access tools for coordinating data entry: EpiData, TeamViewer and Dropbox [5]. EpiData was the software used for data entry and data appending/merging [8]. In EpiData language, a variable is referred to as ‘field’ and a questionnaire (set of fields) as ‘record’. EpiData triplet files used for data entry include a QES (QuEStionnaire) file containing data structure, REC (RECord) file where data entry is carried out and CHK (CHecK) file with data entry checks. TeamViewer was used for remote training and troubleshooting. Dropbox was used for near-real-time file sharing, storing and automatic back-up (Box 1).

#### Data entry tool

The data entry tool included a data documentation sheet (codebook containing the plan for data entry) and EpiData database (consisting of QES, REC, CHK triplet files), separately prepared for household and individual level questionnaires. After developing the first draft of the data entry tool at The Union, New Delhi in March 2016 (NB and HDS), it was pre-tested at Chennai, Tamil Nadu, for suggestions to reduce possible data entry errors (through data entry checks) and number of key strokes per record (April 2016). In the final tool, there were 17 fields in the household level and 160 fields in the individual level REC file. Each REC file was encrypted with a password. The key data entry checks have been summarized in Box 2.

#### Setting up of data entry

The data entry tool was shared in a Dropbox folder. Each district or zone had a separate Dropbox shared folder. Codes for districts/zones (‘value’ and ‘value labels’ in EpiData language), already available as a Microsoft Excel database, were imported as an external label block (*site.rec* in supplementary online material). Among the researchers, this Dropbox shared folder was accessible only to the responsible officials, respectively, from The Union (HDS) and Cancer Institute (DPS).

Half-day training for data entry operators (DEOs) was conducted by the Cancer Institute (WIA) in Tamil Nadu with remote support from The Union. The Union trained DPS over Teamviewer and DPS in turn trained all the DEOs in person. Double data entry was carried out between May 2016 and August 2016 at a single site in Tamil Nadu. We planned to enter data districtwise (*n* = 31) and zonewise (*n* = 15): thus, 46 Dropbox shared folders containing copies of the data entry tool were prepared. It was distributed among 10 DEOs – each DEO was provided access only to those districts/zones’ Dropbox shared folder for which s/he was allocated to enter data. Two DEOs simultaneously worked at one district/zone and completed the double data entry independently (anonymized data). We used this sectoral approach (10 DEOs completing data entry for five districts/zones at a time) to complete data entry for all 31 districts and 15 zones. A validation report for data entry errors was simultaneously prepared and a final household and individual level REC file was prepared for each district/zone after making corrections. Data entry was near real time and therefore, was monitored remotely. As and when required, remote trouble-shooting from The Union, New Delhi, was performed using TeamViewer.

All the final 46 (31 districts and 15 zones) household level REC files were combined using the function ‘append’ in EpiData analysis. Similarly all the final 46 individual level REC files were appended. The appended household level REC file was combined with the appended individual level REC file using the function ‘merge’. The unique identifier provided to each household was entered in both household level and individual level REC files. This helped us to link the respective households with their respective household members while merging the dataset.

## Results

Of 32,945 households, there were 111,363 individuals ≥15 years of age.

### Data entry errors – quality assured data entry

The proportion of records with data entry errors, across districts/zones, ranged from 0% to 27% and 0% to 64% in household level and individual level REC files, respectively. The average error was 4% and 24%, respectively. The proportion of fields with data entry errors, across districts/zones, ranged from 0% to 8% for household level REC files and 0% to 6% for individual level REC files. The average error was 0.5% and 0.7%, respectively (Table 1). These are the errors that would have gone unnoticed if single entry was performed. Double data entry and validation helped identify and correct these errors.

**Table 1.** Data entry errors identified during validation of double entered data: Tamil Nadu Tobacco Survey (TNTS), India (2015–16)*.

	Household level records		Individual level records	
District/Zone	Number of records entered (% with data entry error)	Number of fields entered (% with data entry error)	Number of records entered (% with data entry error)	Number of fields entered (% with data entry error)
**Tamil Nadu districts except Chennai**				
Ariyalur	314(27)	6023(2)	1038(64)	168,156(2)
Coimbattore	1643(1)	31,217(0)	5157(8)	835,434(0)
Cuddalore	1077(2)	20,463(0)	3765(56)	609,930(1)
Dharmapuri	601(1)	11,419(0)	2213(10)	35,866(0)
Dindigul	922(3)	17,518(8)	3057(14)	495,234(0)
Erode	1018(1)	19,342(0)	3222(0)	521,964(0)
Kancheepuram	2303(1)	43,757(0)	6683(8)	1,082,646(0)
Kanyakumari	808(1)	15,352(0)	2760(9)	447,120(0)
Karur	552(2)	10,488(0)	1794(12)	290,628(6)
Krishnagiri	702(0)	13,338(0)	2638(17)	427,356(0)
Madurai	1321(5)	25,099(0)	4316(36)	699,192(1)
Nagapattinam	771(2)	14,649(0)	2411(17)	390,582(1)
Nilgiris	388(7)	7372(1)	1242(44)	201,204(1)
Namakkal	835(2)	15,865(0)	2607(33)	422,334(1)
Perambalur	234(6)	4446(1)	779(53)	126,198(1)
Pudukottai	616(4)	11,704(0)	2325(35)	376,650(1)
Ramanathapuram	474(5)	9006(1)	1907(21)	308,934(0)
Salem	1495(7)	28,405(1)	5116(37)	828,792(1)
Sivaganga	470(5)	2930(0)	1853(25)	300,186(0)
Tiruchirapalli	1371(8)	26,049(1)	4948(57)	801,576(2)
Theni	475(3)	9025(0)	1869(38)	302,778(1)
Tirunelveli	1467(1)	27,873(0)	4716(12)	763,992(0)
Thanjavur	888(1)	16,872(0)	3431(11)	555,822(6)
Thoothukudi	788(2)	14,972(0)	2555(25)	413,910(0)
Tiruvallur	1590(4)	30,210(0)	5267(19)	853,254(0)
Tiruppur	1078(2)	20,482(0)	3530(25)	571,860(0)
Tiruvarur	493(2)	9367(0)	1926(31)	312,012(1)
Tiruvannamalai	1004(8)	19,076(1)	3634(26)	588,708(1)
Vellore	1435(3)	27,265(0)	5722(11)	926,964(0)
Villupuram	1423(5)	27,037(1)	4962(26)	803,844(0)
Virudhunagar	868(2)	16,492(0)	2864(15)	463,968(0)
**Chennai zones**				
Tiruvottiyur	249(3)	4731(0)	759(26)	122,958(0)
Manali	244(0)	4636(0)	734(14)	118,908(0)
Madhavaram	218(1)	4142(0)	730(19)	118,260(0)
Tondiarpet	231(1)	4389(0)	757(10)	122,634(0)
Royapuram	239(3)	4541(0)	785(21)	127,170(0)
Tiruvika nagar	219(4)	4161(0)	735(16)	119,070(0)
Ambattur	238(5)	4522(1)	750(40)	121,500(1)
Annanagar	229(2)	4351(0)	730(15)	118,260(0)
Teynampet	235(3)	4465(0)	789(7)	127,818(0)
Kodambakkam	232(9)	4408(1)	744(45)	120,528(1)
Valasaravakkam	226(7)	4294(1)	672(16)	108,864(0)
Alandur	209(0)	3971(0)	739(8)	119,718(0)
Adyar	280(8)	5320(1)	786(33)	127,332(1)
Perungudi	228(5)	4332(1)	692(15)	112,104(0)
Sholinganallur	247(2)	4693(0)	708(41)	114,696(0)

*Total households 32,945; total respondents/individuals 111,363

### Data entry time – efficient data entry

**T**he median (inter-quartile range) time taken for double data entry for a single household level and individual level questionnaire was 30 (24, 40) s and 86 (64, 126) s, respectively (Table 2).

**Table 2.** Median (inter-quartile range) time (in seconds) taken for double data entry of one questionnaire: Tamil Nadu Tobacco Survey (TNTS), India (2015–16)*.

Name of the district/zone	One household level record (a)	One individual level record (b)
**Tamil Nadu**	**30 (24, 40)**	**86 (64, 128)**
**Tamil Nadu districts except Chennai**		
Ariyalur	58 (46, 78)	196 (154, 264)
Coimbattore	26 (22, 34)	66 (52, 92)
Cuddalore	36 (32, 44)	118 (90, 165)
Dharmapuri	30 (26, 40)	84 (68, 114)
Dindigul	28 (26, 34)	62 (50, 82)
Erode	28 (24, 36)	76 (32, 94)
Kancheepuram	24 (22, 30)	74 (60, 94)
Kanyakumari	40 (34, 56)	102 (78, 152)
Karur	32 (26, 42)	82 (62, 124)
Krishnagiri	22 (20, 28)	68 (56, 88)
Madurai	36 (30, 48)	104 (84, 164)
Nagapattinam	24 (20, 30)	82 (68, 112)
Nilgiris	40 (32, 54)	146 (112, 236)
Namakkal	32 (26, 48)	120 (94, 176)
Perambalur	52 (44, 74)	160 (110, 242)
Pudukottai	26 (22, 34)	130 (86, 208)
Ramanathapuram	33 (28, 42)	84 (66, 118)
Salem	36 (30, 58)	110 (82, 158)
Sivaganga	40 (32, 54)	98 (76, 132)
Tiruchirapalli	36 (30, 48)	112 (82, 152)
Theni	40 (32, 50)	128 (102, 170)
Tirunelveli	26 (22, 36)	72 (58, 108)
Thanjavur	36 (32, 46)	90 (74, 132)
Thoothukudi	32 (28, 40)	98 (80, 128)
Tiruvallur	30 (26, 36)	62 (52, 84)
Tiruppur	24 (20, 34)	82 (64, 116)
Tiruvarur	32 (26, 38)	86 (68, 118)
Tiruvannamalai	40 (24, 60)	50 (34, 76)
Vellore	22 (20, 30)	70 (56, 96)
Villupuram	30 (24, 36)	84 (62, 120)
Virudhunagar	32 (28, 38)	74 (60, 96)
**Chennai zones**		
Tiruvottiyur	26 (23, 34)	76 (56, 118)
Manali	24 (20, 28)	66 (50, 94)
Madhavaram	22 (20, 28)	68 (54, 88)
Tondiarpet	22 (20, 30)	64 (48, 90)
Royapuram	26 (22, 38)	62 (50, 94)
Tiruvika nagar	24 (22, 30)	56 (46, 72)
Ambattur	39 (34, 50)	138 (118, 184)
Annanagar	24 (22, 30)	72 (58, 100)
Teynampet	24 (22, 30)	64 (54, 83)
Kodambakkam	44 (36, 54)	158 (128, 214)
Valasaravakkam	24 (20, 32)	74 (56, 114)
Alandur	24 (20, 26)	58 (46, 74)
Adyar	24 (20, 34)	80 (62, 108)
Perungudi	26 (22, 36)	88 (67, 128)
Sholinganallur	32 (26, 44)	73 (54, 100)

*Total households 32,945; total respondents/individuals 111,363

### Data inconsistency

Despite checks during data entry and double data entry/validation, some inconsistency in entered data may occur, which can be explained by errors during data collection. We checked for the same for two key tobacco use indicators: current and past tobacco use. Among interviewed individuals, for current tobacco use, we identified four records with current tobacco use not available or not recorded and 138 records (0.1%) with data inconsistency (current tobacco use status was ‘no’, but current tobacco use status was ‘yes’ as per the information collected under various types of tobacco and vice versa). For past tobacco use, among eligible interviewees (after excluding those with current tobacco use), we identified 110 records (0.1%) with data inconsistency (past tobacco use was ‘yes’, but past tobacco use status was ‘no’ as per the information collected under various types of tobacco).

## Discussion

We used innovative open access technology for data entry of a large state-wide tobacco survey in India with minimal funding support (under 18,000 USD). Data entry was efficient considering it took less than 2 min to double-enter a large questionnaire as in TNTS. Data entry was quality-assured considering a significant amount of data entry errors was identified and corrected. Data inconsistencies were negligible for the key tobacco use indicators.

Large surveys like this provide well-represented data for health advocates to inform policymakers to advance tobacco control that can be used at state, district and even sub-district level. The data from this survey will also reveal inter-district variations in tobacco use prevalence, which is limited in national surveys like GATS, and also improve our understanding of the nature of diversity of tobacco addiction, and the drivers of the epidemic like prevalence by age, household expenditure on tobacco products, and age of initiation, which will aid policymakers in devising better strategies for tobacco control. Considering the policy implications mentioned above, such data have to be of high quality.

The variation in average percentage error was expected depending on the unit of analysis (record or field) and on the type of REC file, whether individual or household level. When we compared the percentage errors during validation (either with fields or with records as the unit of analysis) across household level and individual level REC files, it was expected that the average error would be higher for individual level REC files. The reason for this was the large number of fields in individual level REC file (*n* = 160) compared to household level REC file (*n* = 17). Average percentage errors were higher if the unit of analysis was records (compared to fields) because even when a single field in the record had an error it would be counted as that record having a data entry error.

**Figure 2.**
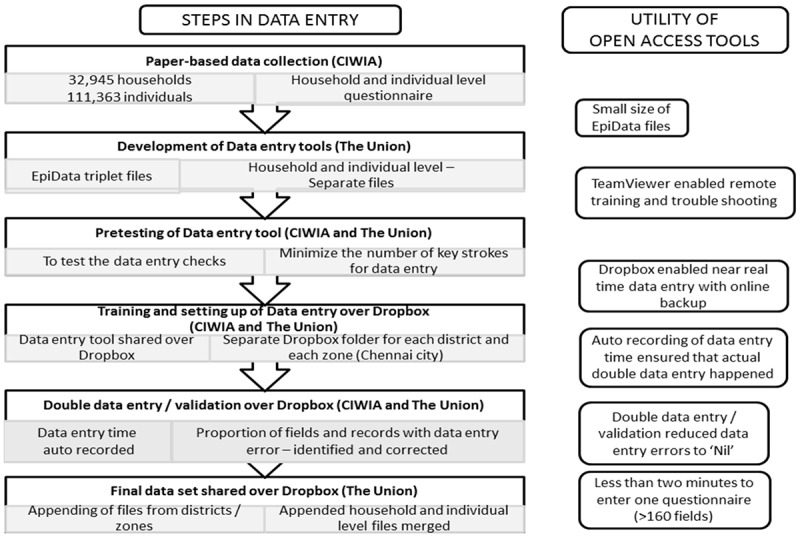
Steps in data entry using open access tools and their utility in Tamil Nadu Tobacco Survey (TNTS), India (2015–16). CIWIA: Cancer Institute, Women’s India Association, Adyar, Chennai, Tamil Nadu, India; The Union: International Union Against Tuberculosis and Lung Disease (The Union), South-East Asia Office, New Delhi, India.

The steps in data entry process and the utility of using open access tools have been summarized in Figure 2. It is worth noting that double data entry of 100,000 records, each containing more than 160 fields took less than 2 min per questionnaire. This efficiency was made possible through appropriate number of checks and minimal number of key strokes required to enter one questionnaire. Most of the data entry errors were identified and corrected. Double data entry and validation was performed, which is considered the gold standard for reducing data entry errors [3,4]. Auto-recording of time taken to enter each record (this cannot be edited) ensured that actual double data entry happened and the DEO did not ‘copy and paste’ single entered data. In the event of single data entry followed by ‘copy and paste’, the data entry time would have exactly matched for each record.

Data entry in a shared dropbox folder allowed real-time monitoring of the process, when connected over the internet. However, internet connectivity was not essential for data entry as Dropbox could be accessed in offline mode. Use of Dropbox also helped in ensuring that all data were backed up online and eliminated the fear of data loss. We believe that any data inconsistencies reported were due to errors during data collection. In future, introduction of additional data entry checks apart from those described in Box 2, may help identify these data collection errors. These could have been eliminated altogether using electronic data capture during the interview itself using mobile hand-held computers (tablets or smartphones), with data checks and skip patterns incorporated. However, this could not be planned in TNTS as the entire 18000 USD was not available and assured at the beginning of the survey. Smartphones are relatively inexpensive (basic tablets can cost 60 USD): this may be considered in future, subject to availability of budget.

Considering the small size of EpiData files, this model of data entry can be replicated in other resource-constrained settings where internet connectivity is poor.

‘Automatic Forms Processing’ is a possible alternative to double entry, a method by which data collected can be ‘automatically’ entered by scanning, and converting it into an electronic format through techniques such as ‘Optical Mark Recognition’ or ‘Intelligent Character Recognition’ [9]. This would also require relatively expensive equipment and computer expertise that are often not available in resource-limited settings.

## Conclusion

In this large sub-national tobacco survey from India involving paper-based data from more than 100,000 respondents, we used open access tools for near-real-time quality assured (with adequate checks, double entry and validation) and efficient data entry with remote monitoring and trouble-shooting.

**Box 1.** Description of open access tools Dropbox and TeamViewer ^5.^

What is Dropbox?
Dropbox is a file hosting service operated by Dropbox Inc. that offers cloud storage and file synchronization. Dropbox uses a ‘Freemium’ business model, where users are offered a free account with a set storage size (2 gigabytes in this case) and paid subscriptions for accounts with more capacity. Dropbox allows users to create a special folder on each of their computers, which Dropbox then synchronises so that it appears to be the same folder (with the same contents) regardless of the computer it is viewed on. Files placed in this folder are also accessible through a website and mobile phone applications. Such folders can be shared with others for mutual access. More information at www.dropbox.com
What is TeamViewer?
TeamViewer is a secure software package for remote control, desktop sharing, online meetings, web conferencing and file transfer between computers. TeamViewer is a tool that makes it very easy to set up and use a Virtual Private Network connection that lets you take complete control of another computer from your own computer via internet. It enables two-way connections in which users can flip control back and forth. While TeamViewer is proprietary, it is free for non-commercial purposes. More information at www.teamviewer.com

**Box 2.** Salient data entry checks incorporated to prevent data entry errors in Tamil Nadu Tobacco Survey (TNTS), India (2015–16).

**I. Both in household and individual level CHK files**
i. Data entry time for each record was auto recorded as an auto generated field. This prevented the data entry operator from copying the single entered record and therefore, feigning double data entry. ii. Each household and individual under an urban and rural area of a district or slum and non-slum area of a zone of Chennai had a unique identifier. iii. Unique identifier was a derived field and was auto generated after entering the individual variables. Generation of unique identifier was made compulsory before moving to next record.
**II. Household level CHK file**
i. Number of persons >=15 years cannot be greater than total household members. ii. Among number of persons≥15 years, sum of males and females should match the total household members.
**III. Individual level CHK file**
i.. If the questionnaire was not filled for an adult ≥15 years in the household, then after entering ‘no’ for the question “whether questionnaire was filled”, all other questions in the record were marked as ‘not applicable’ and the cursor after saving the record went to next record for data entry. ii. If question on current and past smoking status were entered as ‘no’, then all the questions related to tobacco use were marked as “not applicable” and the cursor went to questions targeted on tobacco non-users iii. If current smoker was ‘yes’, then past smoker question was auto filled as ‘not applicable’; and questions on age of stopping various tobacco products were also auto filled as ‘not applicable’. iv. If past smoker was ‘yes’, then all questions pertaining to current smoking were marked as ‘not applicable’ v. If occupation was either student/unemployed/homemaker/retired/missing, then work place related questions were auto entered as ‘not applicable’.

## Supplementary Material

Supplementary material

## Paper context

In GATS-India (2009-2010), data collection and entry were combined into a single step by the use of hand-held computer devices with built-in checks for quality control. In a resource-constrained setting, we used open access tools for efficient and quality-assured data entry of paper-based data collected from a large sub-national tobacco survey in Tamil Nadu, India (2015-2016). The tools described and shared here may be adapted and used by researchers in other resource-constrained settings.
