# Supplementary material for: Open access tools for quality-assured and efficient data entry in a large, state-wide tobacco survey in India
Source: Glob Health Action. 2017 Nov 2;10(1):1394763. doi: 10.1080/16549716.2017.1394763 (PMC5678223; doi:10.1080/16549716.2017.1394763)
Supplement: Supplementary material [file ZGHA_A_1394763_SM3378.pdf]

Supplementary material:

“Open access tools for quality-assured and efficient data entry in a large, state-wide tobacco survey in India” Shewade et al., Global HealthAction

Documents containing questionnaire used for data collection; data documentation sheet (codebook) and EpiData data entry tools (EpiData triplet files (QES, REC and CHK)).

1. Data collection tool – TNTS tool
2. Data documentation sheet for data entry of household level data
3. Data documentation sheet for data entry of individual level data
4. EpiData triplet files for data entry of household level data: HHTN.CHK
5. HHTN.QES
6. HHTN.REC
7. EpiData triplet files for data entry of individual level data: INDITN.CHK
8. INDITN.QES
9. INDITN.REC
10. External label block for name of the district / zone: SITE.CHK
11. SITE.REC

1.TNTS tool

Tamil Nadu Tobacco Survey (TNTS)
Household Questionnaire (Respondent should be above 18 YEARS)

Address: **Zone:**
S.No :

An important survey of adult tobacco use behavior is being conducted by the Cancer Institute throughout Tamil Nadu and your household has been selected toparticipate.All houses selected were chosen from a scientific sample and it is very important to the success of thisproject that each participates in the survey. All information gathered will be kept strictly confidential. Ihave a few questions to find out who in your household is eligible to participate.

1. How many persons live in this household? ______
2. How many of these household members are 15 years of age or older?_____ Male ______, Female _______
3. List all the members above 15 years starting from the oldest

S.No.	Name	Gender	Age	Occupation	Education	Mobile no.

4. Household Items:

1. Electricity ☐ 2. Flush toilet ☐ 3.Fixed telephone ☐ 4. Mobile ☐
5. Television ☐ 6. Radio ☐ 7. Refrigerator ☐ 8. Car ☐
9. Two wheeler ☐ 10.Washing machine ☐

Need to call all the eligible members of the family and fix appointment or interview over phone. For each individual use a separate form and take consent.
Consent from respondent, if below 18 years both from parent and respondent

I am working/studyingin____________________[Name of Organization]. Cancer Institute is doing a survey about tobacco use in Tamil Nadu. ___________ is one of the collaborating organization/institution for this survey. This information will be used for planning public health programs.

Your household have been selected at random. Your responses are very important to us and the community. These answers will represent many other persons. The interview will last approximately 20 minutes. Your participation in this survey is entirely voluntary. The information that you will provide will be kept strictly confidential and the name will not be identified by your responses. There will not be any direct benefits to you, but the results will help the Government and other Non Governmental Organizations plan public health programs for the state.

We will leave the necessary contact information with you. If you have any questions about this survey or your rights as a participant, you can contact the telephone numbers listed. If you agree to participate in this survey, we will conduct a private interview with you.

1.TNTS tool

Individual Questionnaire

Do you agree to participate?
Adult/Minor respondent: 1. Yes 2. No. If minors, PARENT/GUARDIAN: 1.Yes 2. No

----

1. **Do you *currently* use Tobacco** 1.Yes 2. No

2. **In the *past*, have you used Tobacco ?** 1.Yes 2. No

If the answer is 1 or 2 for any of the above two questions go to 3rd question or else go to question No 21.

3. Smoking and Chewing Habits

Type	Name of the Tobacco/ Alcohol	Current (1) Past (2)	Frequency		Age Started (Year)	(past user) Age/year Stopped	Duration of use
			Daily	Weekly			
**Smoking**							
Cigarettes (Filtered/Non-filtered)							
Bidi							
Cigarette							
Chutta/Cigar/Cheroot							
Others (specify)							
**Chewing**							
Tobacco alone							
AN or BQ, AN +BQ							
AN +BQ+Tobacco							
AN and Tobacco							
Pan with tobacco							
Others							
**Snuffing**							
By Mouth							
By Nose							
**Alcohol__________**							

4. How soon after you wake up do you usually have your first smoke/chew/snuff?
1. Within 5 minutes, **2.** 6 to 30 minutes, **3.** 31 to 60 minutes,**4.** More than 60 minutes

5. When you bought tobacco last time, how many cigarettes/BIDIS/Sackets did you buy?
____
If it is a pack, how many sticks were there in the packet? ______________

6. In total, how much money did you pay for this purchase? _________

7. **Expense :No _____ X Rs _____ X 30 (Days) = ______ X 12 X______ (No. of years)= ____________**

1.TNTS tool

8. Are you concerned about your tobacco use? 1.Yes 2. No

9. Have you visited a doctor or other health care provider in the past 12 months? 1.Yes 2. No

10. During any visit to a doctor or health care provider in the past 12 months, were you asked if you use Tobacco? 1.Yes 2. No

11.During any visit to a doctor or health care provider in the past 12 months, were you advised to quit Tobacco use? 1.Yes 2. No

12. In the last 30 days, have you noticed any warning on the tobacco product you use? 1.Yes 2. No

13. In the last 30 days, the warning labels on tobacco products led you to think about quitting? 1.Yes 2. No

14.During the past 12 months, did you use any of the following methods to try to stop using tobacco?

1. Counseling ☐ **2.**Nicotine replacement therapy ☐ **3**. Medications ☐

4. Switching to alternate tobacco ☐ **5**. A quit line ☐ **6**.Traditional medicines ☐

7. Quit on my own ☐ 8. No attempt ☐ 9. Others ________________

15.If you have quit completely, specify the method you found effective. ___________________

16.Thinking about the last time you tried to quit, how long did you stop using tobacco? _____

1. MONTHS ☐ **2.**WEEKS ☐ **3.** DAYS ☐ **4.**LESS THAN 1 DAY (24 HOURS**)** ☐ **5**. No change ☐

17. Which of the following best describes your thinking about quitting tobacco?

1. WITHIN ONE MONTH ☐ **2**. WITHIN 12 MONTHS ☐ **3.** QUIT SOMEDAY, BUT NOT NEXT 12 MONTHS ☐

4. DON'T KNOW ☐ **5.** NOT INTERESTED IN QUITTING ☐

☐

If you are less than 18 years

18. when you purchased tobacco in the past 30 days were you refused tobacco? 1. Yes ☐ 2. No ☐

If Chewers

19. Did you find it difficult to purchase tobacco in the past 30 days? 1. Yes ☐ 2. No ☐

20**. What was the price before and after the ban for the tobacco you usually purchase?**

1. Brand Name______________, Before: Rs ____________, After: Rs____________

Ask these questions for non-users also

21. Do you know the harmful effects of passive smoking? **1. Yes** ☐ **2. No** ☐

22. Which of the following best describes the rules about smoking inside of your home?

1. ALLOWED ☐ **2**. NOT ALLOWED, BUT EXCEPTIONS ☐ **3**. NEVER ALLOWED ☐

4. NO RULES ☐ **5**. DON'T KNOW ☐

1.TNTS tool

23. How often does *anyone* smoke inside your home? **1. Yes** ☐ **2. No** ☐
If yes,
1. DAILY ☐ **2.**WEEKLY ☐ **3**. MONTHLY ☐

4.LESS THAN MONTHLY ☐ **5.**NEVER ☐ 6.DON'T KNOW ☐

24. Which of the following best describes the indoor smoking policy where you work?
1. ALLOWED ANYWHERE ☐ **2.**ALLOWED ONLY IN SOME INDOOR AREAS ☐ **3.** ONLY IN OUT DOOR AREAS ☐

4. NOT ALLOWED ANYWHERE ☐ **5.**THERE IS NO POLICY ☐ **6.**DON'T KNOW ☐

25. During the past 30 days, did anyone smoke in indoor areas where you work?
1. YES ☐ **2.**NO ☐ **3.**DON'T KNOW ☐

26. Based on what you know or believe, does breathing other people's smoke cause serious illness in Non smokers?
1. YES ☐ **2.**NO ☐ **3.**DON'T KNOW ☐

27. Based on what you know or believe does tobacco cause the following…
1. Stroke ☐ **2**. Heart attack ☐ **3.** Cancer ☐
4. Cause serious illness ☐ 5. Infertility/impotence ☐ 6. Don't know ☐

28. In the last 30 days, have you seen any information about the danger use of tobacco or that encourages quitting of tobacco products?
1. Television ☐ **2.** Radio ☐ **3.**News papers ☐ **4.** Billboards ☐
5. Tobacco packs ☐ 6. Movies ☐ 7. Theatres ☐ 8. Not seen ☐

29. In last 30 days, have you seen any advertisement that encourages or promoting tobacco products?
1. Television ☐ **2.** Radio ☐ **3.**News papers ☐ **4.** Billboards ☐
5.Tobacco packs ☐ 6. Movies ☐ 7. Theatres ☐ 8. Not seen ☐

30. Do you suggest any measures for curbing the use of tobacco?

Thank you for your participation.

Signature

2.

HOUSEHOLD – DATA Documentation SHEET

S.No	Field name	Field label	Field type	Field length	Field values	Value labels	Comments
1	idhouse	Unique id for the household	S	11			Unique identifier. Noenter field. Will get autogenerated. idhouse=distcode+hhno
2	distcode	District code	S	6			No enter field. Autogenerated from distarea
3	distarea	Name of the district including area	S	40			Select from the options depending on the name of the district and area (urban or rural). For Chennai select depending on the name of the zone and area (slum or non slum)
4	hhno	Household serial number	S	4	`1-2999		Enter 2001,2002 onwards if hhno is missing or non unique
5	Q1	Number of household members/persons	I	2	1-20,99		Enter 99 if the data is not recorded
6	Q2	Number of household members of age 15 years or older	I	2	1-20,99		Enter 99 if the data is not recorded
7	Q2a	Number of household male members of age 15 years or older	I	2	1-20,99		Enter 99 if the data is not recorded
8	Q2b	Number of household female members of age 15 years or older	I	2	1-20,99		Enter 99 if the data is not recorded
9	Q3a	Electricity	I	1	1<br>2	Yes<br>no	
10	Q3b	Flush toilet	I	1	1<br>2	Yes<br>no	
11	Q3c	Fixed telephone	I	1	1<br>2	Yes<br>no	
12	Q3d	Mobile	I	1	1<br>2	Yes<br>no	
13	Q3e	Television	I	1	1<br>2	Yes<br>no	
14	Q3f	radio	I	1	1<br>2	Yes<br>no	
15	Q3g	Refrigerator	I	1	1<br>2	Yes<br>no	
16	Q3h	Car	I	1	1<br>2	Yes<br>no	
17	Q3i	Two wheeler	I	1	1<br>2	Yes<br>no	
18	Q3j	Washing machine	I	1	1<br>2	Yes<br>no	

3.

INDIVIDUAL – DATA Documentation SHEET

S.No	Field name	Field label	Field type	Field length	Field values	Value labels	Comments
1	idindi	Unique id for the individual / record	S	17			Unique id. Noenter field. Will be auto generated from dstcode and indino
2	idhouse	Household id	S	11			Key field used to link individual record with household record. Noenter field. Will get autogenerated. idhouse=distcode+hhno
3	distcode	District code	S	6			No enter field. Autogenerated from distarea
4	distarea	Name of the district including area	S	40			Select from the options depending on the name of the district and area (urban or rural). For Chennai select depending on the name of the zone and area (slum or non slum)
5	hhno	Household serial number	I	4	`1-2999		Enter 2001,2002 onwards if hhsno is missing
6	indino	Individual participant's identification number	S	10<br>####.####a/b/c/d	1.1 – 2000.9999		In case of missing individual use respective missing household individual number (use the last household number and add a, b, c for those household members for whom interview couldn't be taken
7	gender	Participant's gender	I	1	1<br>2<br>3<br>9	M<br>F<br>TG<br>Missing	
8	age	Participant's age	I	3	15-110<br>999		enter 999 if the data is not recorded
9	occ	Participant's occupation	I	1	0<br>1<br>2<br>3<br>4<br>5<br>6<br>7<br>8<br>9	Student<br>Unemployed<br>Homemaker<br>Daily Wage<br>Driver<br>Private job<br>Govt job<br>Self emp<br>Retired<br>Not record	
10	edn	Participant's	I	1	1	No formal school	

INDIVIDUAL – DATA Documentation SHEET

		education			2<br>3<br>4<br>5<br>9	Primary<br>Sec<br>Hr Sec<br>Degree or higher<br>Not recorded	
	phno	Individual phone number	I	10			Do not enter 0 or +91 before the number. Enter 9999999999 for missing
11	filled	Was questionnaire filled?	I	1	1<br>2	Yes<br><br>No	If no, then the reason for not filling must be filled and the fields after that must be marked as not applicable<br>If yes, then the reason should be not applicable and cursor should move to next field
12	reason	Why was the questionnaire not filled			1<br>2<br>8<br>9	Not available<br>Did not consent<br>Not applicable<br>reason not recorded	If filled is 'no', then not applicable should not be filled here.
13	Q1	Do you currently use tobacco?	I	1	1<br>2<br>8<br>9	Yes<br>No<br>Not applicable<br>Not recorded	If q1=1 then q2=8 and cursor will go to q3sma1
14	Q2	In the past have you used tobacco?	I	1	1<br>2<br>8<br>9	Yes<br>No<br>Not applicable<br>Not recorded	If q1 and q2 are no then fields below should be filled as not applicable and cursor should go to q21
	Q3sma	Whether user of cigarette	I	1	1<br>2	Yes<br>No	Yes if respective row in Q3 table has been filled. This is not to be included during analysis, variable created only to assist efficient data entry
15	Q3sma1	Name of the tobacco smoking cigarettes (filtered/non-filtered)	S	15			Mention not applicable if required
16	Q3sma2	Cigarettes (filtered/non-filtered) smoking Current/Past	I	1	1<br>2<br>8<br>9	Current<br>Past<br>Not applicable<br>Not recorded	
17	Q3sma3d	Cigarettes (filtered/non-filtered) daily frequency	I	2	1-70<br>98<br>99		enter 99 if the data is not recorded, 98 if not applicable<br>Enter Q3sma3d or q3sma3w (any one) whichever is

INDIVIDUAL – DATA Documentation SHEET

							applicable
18	Q3sma3w	Cigarettes (filtered/non-filtered) weekly frequency	I	3	1-490<br>998<br>999		enter 999 if the data is not recorded, 998 if not applicable
19	Q3sma4	Cigarettes (filtered/non-filtered) age started in	I	2	3-97<br>98<br>99		enter 99 if the data is not recorded, 98 if not applicable
20	Q3sma5	Cigarettes (filtered/non-filtered) age past user stopped at	I	3	3-110<br>998<br>999		enter 999 if the data is not recorded, 998 if not applicable
21	Q3sma6	Cigarettes (filtered/non-filtered) duration of use (completedyear)	I	2	0-97<br>98<br>99		No enter field<br>98 is not applicable
	Q3smb	Whether user of bidi	I	1	1<br>2	Yes<br>No	Yes if respective row in Q3 table has been filled. This is not to be included during analysis, variable created only to assist efficient data entry
22	Q3smb1	Name of the tobacco smoking bidi brand	S	15			Mention not applicable if required
23	Q3smb2	bidi smoking Current/Past	I	1	1<br>2<br>8<br>9	Current<br>Past<br>Not applicable<br>Not recorded	
24	Q3smb3d	Bidi smoking daily frequency	I	2	1-70<br>98<br>99		enter 99 if the data is not recorded, 98 if not applicable<br>Enter Q3smb3d or q3smb3w (any one) whichever is applicable
25	Q3smb3w	Bidi smoking weekly frequency	I	3	1-490<br>998<br>999		enter 999 if the data is not recorded, 998 if not applicable
26	Q3smb4	Bidi smoking age started in	I	2	3-97<br>98<br>99		enter 99 if the data is not recorded, 98 if not applicable
28	Q3smb5	Bidi smoking age past user stopped at	I	3	3-110<br>998<br>999		enter 999 if the data is not recorded, 998 if not applicable
29	Q3smb6	Bidi smoking duration of use	I	2	0-97<br>98		No enter field<br>98 is not applicable

INDIVIDUAL – DATA Documentation SHEET

					99		
	Q3smc	Whether user of chutta/cigar/chi root	I	1	1<br>2	Yes<br>No	Yes if respective row in Q3 table has been filled. This is not to be included during analysis, variable created only to assist efficient data entry
30	Q3smc1	Name of the tobacco smoking chutta/cigar/ch eroot brand	S	15			Mention not applicable if required
31	Q3smc2	chutta/cigar/ch eroot smoking Current/Past	I	1	1<br>2<br>8<br>9	Current<br>Past<br>Not applicable<br>Not recorded	
32	Q3smc3d	chutta/cigar/ch eroot smoking daily frequency	I	2	1-70<br>98<br>99		enter 99 if the data is not recorded, 98 if not applicable.<br>Enter Q3smc3d or q3smc3w (any one) whichever is applicable
33	Q3smc3w	chutta/cigar/ch eroot smoking weekly frequency	I	3	1-490<br>998<br>999		enter 999 if the data is not recorded, 998 if not applicable
34	Q3smc4	chutta/cigar/ch eroot smoking age started in	I	2	3-97<br>98<br>99		enter 99 if the data is not recorded, 98 if not applicable
35	Q3smc5	chutta/cigar/ch eroot smoking age past user stopped at	I	3	3-110<br>998<br>999		enter 999 if the data is not recorded, 998 if not applicable
36	Q3smc6	chutta/cigar/ch eoot smoking duration of use	I	2	0-97<br>98<br>99		No enter field<br>98 is not applicable
	Q3smd	Whether user of smoking, others	I	1	1<br>2	Yes<br>No	Yes if respective row in Q3 table has been filled. This is not to be included during analysis, variable created only to assist efficient data entry
37	Q3smd1	Name of the tobacco smoking Othes brand	S	15			Mention not applicable if required
38	Q3smd2	Others smoking Current/Past	I	1	1<br>2<br>8<br>9	Current<br>Past<br>Not applicable<br>Not recorded	

INDIVIDUAL – DATA Documentation SHEET

39	Q3smd3d	Others smoking daily frequency	I	2	1-70<br>98<br>99		enter 99 if the data is not recorded, 98 if not applicable.<br>Enter Q3smd3d or q3smd3w (any one) whichever is applicable
40	Q3smd3w	Others smoking weekly frequency	I	3	1-490<br>998<br>999		enter 999 if the data is not recorded, 998 if not applicable
41	Q3smd4	Others smoking age started in	I	2	3-97<br>98<br>99		enter 99 if the data is not recorded, 98 if not applicable
42	Q3smd5	Others smoking age past user stopped at	I	3	3-110<br>998<br>999		enter 999 if the data is not recorded, 998 if not applicable
43	Q3smd6	Others smoking duration of use	I	2	0-97<br>98<br>99		No enter field<br>98 is not applicable
	Q3cha	Whether user of chewing tobacco, alone	I	1	1<br>2	Yes<br>No	Yes if respective row in Q3 table has been filled. This is not to be included during analysis, variable created only to assist efficient data entry
44	Q3cha1	Name of the chewing tobacco alone	S	15			Mention not applicable if required
45	Q3cha2	Tobacco alone chewing Current/Past	I	1	1<br>2<br>8<br>9	Current<br>Past<br>Not applicable<br>Not recorded	
46	Q3cha3d	Tobacco alone chewing daily frequency	I	2	1-70<br>98<br>99		enter 99 if the data is not recorded, 98 if not applicable.<br>Enter Q3cha3d or q3cha3w (any one) whichever is applicable
47	Q3cha3w	Tobacco alone chewing weekly frequency	I	3	1-490<br>998<br>999		enter 999 if the data is not recorded, 998 if not applicable
48	Q3cha4	Tobacco alone chewing age started in	I	2	3-97<br>98<br>99		enter 99 if the data is not recorded, 98 if not applicable
49	Q3cha5	Tobacco alone chewing age past user stopped at	I	3	3-110<br>998<br>999		enter 999 if the data is not recorded, 998 if not applicable
50	Q3cha6	Tobacco alone chewing	I	2	0-97<br>98		No enter field<br>98 is not applicable

INDIVIDUAL – DATA Documentation SHEET

		duration of use			99		
	Q3chb	Whether user of chewing TOBACCO/AN/PAN	I	1	1<br>2	Yes<br>No	Yes if respective row in Q3 table has been filled. This is not to be included during analysis, variable created only to assist efficient data entry
51	Q3chb1	Name of the chewing TOBACCO/AN/PAN	S	15			Mention not applicable if required
52	Q3chb2	TOBACCO/AN/PAN chewing Current/Past	I	1	1<br>2<br>8<br>9	Current<br>Past<br>Not applicable<br>Not recorded	
52	Q3chb3d	TOBACCO/AN/PAN chewing daily frequency	I	2	1-70<br>98<br>99		enter 99 if the data is not recorded, 98 if not applicable<br>Enter Q3chb3d or q3chb3w (any one) whichever is applicable
53	Q3chb3w	TOBACCO/AN/PAN chewing weekly frequency	I	3	1-490<br>998<br>999		enter 999 if the data is not recorded, 998 if not applicable
54	Q3chb4	TOBACCO/AN/PAN chewing age started in	I	2	3-97<br>98<br>99		enter 99 if the data is not recorded, 98 if not applicable
55	Q3chb5	TOBACCO/AN/PAN chewing age past user stopped at	I	3	3-110<br>998<br>999		enter 999 if the data is not recorded, 998 if not applicable
56	Q3chb6	TOBACCO/AN/PAN chewing duration of use	I	2	0-97<br>98<br>99		No enter field<br>98 is not applicable
	Q3chc	Whether user of chewing Tobacco+Pan	I	1	1<br>2	Yes<br>No	Yes if respective row in Q3 table has been filled. This is not to be included during analysis, variable created only to assist efficient data entry
57	Q3chc1	Name of the chewing Tobacco+Pan	S	15			Mention not applicable if required
58	Q3chc2	Tobacco+Pan chewing Current/Past	I	1	1<br>2<br>8<br>9	Current<br>Past<br>Not applicable<br>Not recorded	
59	Q3chc3d	Tobacco+Pan chewing daily	I	2	1-70<br>98		enter 99 if the data is not recorded, 98 if not

		frequency			99		applicable<br>Enter Q3chc3d or q3chc3w (any one) whichever is applicable
60	Q3chc3w	Tobacco+Pan chewing weekly frequency	I	3	1-490<br>998<br>999		enter 999 if the data is not recorded, 998 if not applicable
61	Q3chc4	Tobacco+Pan chewing age started in	I	2	3-97<br>98<br>99		enter 99 if the data is not recorded, 98 if not applicable
62	Q3chc5	Tobacco+Pan chewing age past user stopped at	I	3	3-110<br>998<br>999		enter 999 if the data is not recorded, 998 if not applicable
63	Q3chc6	Tobacco+Pan chewing duration of use	I	2	0-97<br>98<br>99		No enter field<br>98 is not applicable
	Q3chd	Whether user of chewing Tobacco, others	I	1	1<br>2	Yes<br>No	Yes if respective row in Q3 table has been filled. This is not to be included during analysis, variable created only to assist efficient data entry
64	Q3chd1	Name of the chewing Tobacco, others	S	15			Mention not applicable if required
65	Q3chd2	Tobacco, others chewing Current/Past	I	1	1<br>2<br>8<br>9	Current<br>Past<br>Not applicable<br>Not recorded	
66	Q3chd3d	Tobacco, others chewing daily frequency	I	2	1-70<br>98<br>99		enter 99 if the data is not recorded, 98 if not applicable.<br>Enter Q3chd3d or q3chd3w (any one) whichever is applicable
67	Q3chd3w	Tobacco, others chewing weekly frequency	I	3	1-490<br>998<br>999		enter 999 if the data is not recorded, 998 if not applicable
68	Q3chd4	Tobacco, others chewing age started in	I	2	3-97<br>98<br>99		enter 99 if the data is not recorded, 98 if not applicable
69	Q3chd5	Tobacco, others chewing age past user stopped at	I	3	3-110<br>998<br>999		enter 999 if the data is not recorded, 998 if not applicable
70	Q3chd6	Tobacco, others chewing duration of use	I	2	0-97<br>98<br>99		No enter field<br>98 is not applicable

INDIVIDUAL – DATA Documentation SHEET

	Q3sna	Whether user of snuffing tobacco, by mouth	I	1	1<br>2	Yes<br>No	Yes if respective row in Q3 table has been filled. This is not to be included during analysis, variable created only to assist efficient data entry
85	Q3sna1	Name of the snuffing Tobacco, by mouth	S	15			Mention not applicable if required
86	Q3sna2	Snuffing by mouth Current/Past	I	1	1<br>2<br>8<br>9	Current<br>Past<br>Not applicable<br>Not recorded	
87	Q3sna3d	Snuffing by mouth daily frequency	I	2	1-70<br>98<br>99		enter 99 if the data is not recorded, 98 if not applicable<br>Enter Q3sna3d or q3sna3w (any one) whichever is applicable
88	Q3sna3w	Snuffing by mouth weekly frequency	I	3	1-490<br>998<br>999		enter 999 if the data is not recorded, 998 if not applicable
89	Q3sna4	Snuffing by mouth age started in	I	2	3-97<br>98<br>99		enter 99 if the data is not recorded, 98 if not applicable
90	Q3sna5	Snuffing by mouth age past user stopped at	I	3	3-110<br>998<br>999		enter 999 if the data is not recorded, 998 if not applicable
91	Q3sna6	Snuffing by mouth duration of use	I	2	0-97<br>98<br>99		No enter field<br>98 is not applicable
	Q3snb	Whether user of snuffing tobacco, by nose	I	1	1<br>2	Yes<br>No	Yes if respective row in Q3 table has been filled. This is not to be included during analysis, variable created only to assist efficient data entry
92	Q3snb1	Name of the snuffing Tobacco, by nose	S	15			Mention not applicable if required
93	Q3snb2	Snuffing by nose Current/Past	I	1	1<br>2<br>8<br>9	Current<br>Past<br>Not applicable<br>Not recorded	
94	Q3snb3d	Snuffing by nose daily frequency	I	2	1-70<br>98<br>99		enter 99 if the data is not recorded, 98 if not applicable<br>Enter Q3snb3d or q3snb3w

INDIVIDUAL – DATA Documentation SHEET

							(any one) whichever is applicable
95	Q3snb3w	Snuffing by nose weekly frequency	I	3	1-490<br>998<br>999		enter 999 if the data is not recorded, 998 if not applicable
96	Q3snb4	Snuffing by nose age started in	I	2	3-97<br>98<br>99		enter 99 if the data is not recorded, 98 if not applicable
97	Q3snb5	Snuffing by nose age past user stopped at	I	3	3-110<br>998<br>999		enter 999 if the data is not recorded, 998 if not applicable
98	Q3snb6	Snuffing by nose duration of use	I	2	0-97<br>98<br>99		No enter field<br>98 is not applicable
	Q3ala	Whether user of alcohol	I	1	1<br>2	Yes<br>No	Yes if respective row in Q3 table has been filled. This is not to be included during analysis, variable created only to assist efficient data entry.
99	Q3ala1	Name of the alcohol use	S	15			Mention not applicable if required
100	Q3ala2	Alcohol Current/Past	I	1	1<br>2<br>8<br>9	Current<br>Past<br>Not applicable as non alcoholic (both current and past)<br>Not recorded	
101	Q3ala3d	Alcohol daily frequency	I	2	1-70<br>98<br>99		enter 99 if the data is not recorded, 98 if not applicable<br>Enter Q3ala3d or q3ala3w (any one) whichever is applicable
102	Q3ala3w	Alcohol weekly frequency	I	3	1-490<br>998<br>999		enter 999 if the data is not recorded, 998 if not applicable
103	Q3ala4	Alcohol age started in	I	2	3-97<br>98<br>99		enter 99 if the data is not recorded, 98 if not applicable
104	Q3ala5	Alcohol past user stopped at	I	3	3-110<br>998<br>999		enter 999 if the data is not recorded, 998 if not applicable
105	Q3ala6	Alcohol duration of use	I	2	0-97<br>98<br>99		No enter field<br>98 is not applicable
106	Q4	Post wake up, when do you usually have	I	1	1<br>2<br>3	Within 5 minutes<br>6 to 30 minutes<br>31 to 60 minutes	

		your first smoke/chew/snuff?			4<br><br>8<br>9	More than 60 minutes<br>Not applicable<br>Not recorded	
107	Q5	Number of cigarettes/bidis/ sachets purchased last time	I	2	0-99		Enter 99 if the data is not recorded, 98 if not applicable. If pack is mentioned in the field, then only, the next field is applicable
108	Q5a	If it is a pack, how many sticks were there in the packet?	I	2	1-50		Enter 99 if the data is not recorded, 98 if not applicable
109	Q6	In total, how much money did you pay for this purchase (in INR)	F	####.##	0.01-9999.99		Enter 9999.99 for missing 9999.98 for not applicable
110	Q7	Total expense on this purchase (in INR) till date	F	#########.##	1-999999999.99		Enter 9999.99 for missing 9999.98 for not applicable
111	Q8	Are you concerned about your tobacco use?	I	1	1<br>2<br>8<br>9	Yes<br>No<br>Not applicable<br>Not recorded	
112	Q9	Have you visited a doctor in the past 12 months	I	1	1<br>2<br>8<br>9	Yes<br>No<br>Not applicable<br>Not recorded	
113	Q10	Did the doctor ask if you have used tobacco (in last 12 months)	I	1	1<br>2<br>8<br>9	Yes<br>No<br>Not applicable<br>Not recorded	
114	Q11	Did the doctor advise you to quit tobacco (in last 12 months)	I	1	1<br>2<br>8<br>9	Yes<br>No<br>Not applicable<br>Not recorded	
115	Q12	in last 30 days, did you notice any warning on tobacco product you use?	I	1	1<br>2<br>8<br>9	Yes<br>No<br>Not applicable<br>Not recorded	
116	Q13	In last 30 days, did warning labels led you to think about quitting	I	1	1<br>2<br>8<br>9	Yes<br>No<br>Not applicable<br>Not recorded	
118	Q14.1	In last 12 month did you try Counselling	I	1	1<br>2<br>8	Yes<br>No<br>Not applicable	

INDIVIDUAL – DATA Documentation SHEET

					9	Not recorded	
119	Q14.2	In last 12 month did you try Nicotine replacement therapy	I	1	1<br>2<br>8<br>9	Yes<br>No<br>Not applicable<br>Not recorded	
120	Q14.3	In last 12 month did you try Medications	I	1	1<br>2<br>8<br>9	Yes<br>No<br>Not applicable<br>Not recorded	
121	Q14.4	In last 12 month did you try Switching to alternated tobacco	I	1	1<br>2<br>8<br>9	Yes<br>No<br>Not applicable<br>Not recorded	
122	Q14.5	In last 12 month did you try A quit line	I	1	1<br>2<br>8<br>9	Yes<br>No<br>Not applicable<br>Not recorded	
123	Q14.6	In last 12 month did you try Traditional medicines	I	1	1<br>2<br>8<br>9	Yes<br>No<br>Not applicable<br>Not recorded	
124	Q14.7	In last 12 month did you try Quit on my own	I	1	1<br>2<br>8<br>9	Yes<br>No<br>Not applicable<br>Not recorded	
125	Q14.8	In last 12 month there was no attempt to quit	I	1	1<br>2<br>8<br>9	Yes<br>No<br>Not applicable<br>Not recorded	
126	Q14.9	Any other method to quit	S	40		Mention not applicable if required	
127	Q15	If you have quit completely, specify the method you found effective	I	1	0<br>1<br><br>2<br>3<br><br>4<br>5<br><br>6<br>7<br>8<br>9	Counseling<br>Nicotine replacement<br>Medications<br>Switching to alternate tobacco<br>A quit line<br>Traditional medicines<br>Quit on my own<br>Other<br>Not applicable<br>Not recorded	
128	Q16	How long did you stop using tobacco, since last quit?	I	1	1<br>2<br>3<br>4	Months<br>Weeks<br>Days<br>Less than 1 day	

INDIVIDUAL – DATA Documentation SHEET

					5<br>8<br>9	No change<br>Not applicable<br>Not recorded	
129	Q17	Which of the following best describes about quitting tobacco?	I	1	1<br>2<br>3<br><br>4<br>5<br><br>8<br>9	Within one month<br>Within 12 months<br>Quit someday, but not next 12 months<br>Don't know<br>Not interested in quitting<br>Not applicable<br>Not recorded	
130	Q18	Were you refused while purchasing tobacco in past 30 days? (if less than 18)	I	1	1<br>2<br>8<br>9	Yes<br>No<br>Not applicable<br>Not recorded	
131	Q19	Did you find it difficult to purchase tobacco in past 30 days? (if chewers)	I	1	1<br>2<br>8<br>9	Yes<br>No<br>Not applicable<br>Not recorded	
132	Q20	Brand name of the tobacco that is usually purchased	S	20			Mention not applicable if required
133	Q20a	Price of the tobacco brand (usually purchased) before the ban	F	####.##	0-9999.99		9999.99 for not recorded<br>9999.98 for not applicable
134	Q20b	Price of the tobacco brand (usually purchased) after the ban	F	####.##	0-9999.99		9999.99 for not recorded<br>9999.98 for not applicable
135	Q21	Do you know the harmful effects of passive smoking?	I	1	1<br>2<br>9	Yes<br>No<br>Not recorded	This questionnaire is for users and nonusers of tobacco, both
136	Q22	Which describes the best rule for smoking inside your home?	I	1	1<br>2<br><br>3<br>4<br>5<br>9	Allowed<br>Not allowed, exceptions<br>Never allowed<br>No rules<br>Don't know<br>Not recorded	
137	Q23a	Does anyone	I	1	1	Yes	

		smoke inside your home?			2<br>8<br>9	No<br>Not applicable<br>Not recorded	
138	Q23b	How often does anyone smoke inside your home?	I	1	1<br>2<br>3<br>4<br>5<br>6<br>8<br>9	Daily<br>Weekly<br>Monthly<br>Less than monthly<br>Never<br>Don't know<br>Not applicable<br>Not recorded	Only applicable if q23a is yes
139	Q24	Which best describes the indoor smoking policy where you work?	I	1	1<br>2<br><br>3<br><br>4<br><br>5<br>6<br>8<br>9	Allowed anywhere<br>Allowed in some indoor areas<br>Only in outdoor areas<br>Not allowed anywhere<br>There is no policy<br>Don't know<br>Not applicable<br>Not recorded	
140	Q25	During past 30 days, did anyone smoke in indoor areas where you work?	I	1	1<br>2<br>3<br>8<br>9	Yes<br>No<br>Don't know<br>Not applicable<br>Not recorded	
141	Q26	Does breathing, others smoke cause serious illness in non-smokers?	I	1	1<br>2<br>3<br>8<br>9	Yes<br>No<br>Don't know<br>Not applicable<br>Not recorded	
142	Q27.1	Does tobacco cause Stroke	I	1	1<br>2<br>8<br>9	Yes<br>No<br>Not applicable<br>Not recorded	
143	Q27.2	Does tobacco cause Heart attack	I	1	1<br>2<br>8<br>9	Yes<br>No<br>Not applicable<br>Not recorded	
144	Q27.3	Does tobacco cause Cancer	I	1	1<br>2<br>8<br>9	Yes<br>No<br>Not applicable<br>Not recorded	
145	Q27.4	Does tobacco Cause serious illness	I	1	1<br>2<br>8<br>9	Yes<br>No<br>Not applicable<br>Not recorded	
146	Q27.5	Does tobacco cause	I	1	1<br>2	Yes<br>No	

		Infertility/impotence			8<br>9	Not applicable<br>Not recorded	
147	Q27.6	Does tobacco cause – " don't know" was the answer	I	1	1<br>2<br>8<br>9	Yes<br>No<br>Not applicable<br>Not recorded	
148	Q28.1	Have you seen in last 30 d info about dangers encouraging quit - Television	I	1	1<br>2<br>8<br>9	Yes<br>No<br>Not applicable<br>Not recorded	
149	Q28.2	Have you seen in last 30 d info about dangers encouraging quit - Radio	I	1	1<br>2<br>8<br>9	Yes<br>No<br>Not applicable<br>Not recorded	
150	Q28.3	Have you seen in last 30 d info about dangers encouraging quit - Newspapers	I	1	1<br>2<br>8<br>9	Yes<br>No<br>Not applicable<br>Not recorded	
151	Q28.4	Have you seen in last 30 d info about dangers encouraging quit - Billiboards	I	1	1<br>2<br>8<br>9	Yes<br>No<br>Not applicable<br>Not recorded	
152	Q28.5	Have you seen in last 30 d info about dangers encouraging quit - Tobacco packs	I	1	1<br>2<br>8<br>9	Yes<br>No<br>Not applicable<br>Not recorded	
153	Q28.6	Have you seen in last 30 d info about dangers encouraging quit - Movies	I	1	1<br>2<br>8<br>9	Yes<br>No<br>Not applicable<br>Not recorded	
154	Q28.7	Have you seen in last 30 d info about dangers encouraging quit - Theatres	I	1	1<br>2<br>8<br>9	Yes<br>No<br>Not applicable<br>Not recorded	
155	Q28.8	Have you seen in last 30 d info about dangers encouraging quit - Not seen	I	1	1<br>2<br>8<br>9	Yes<br>No<br>Not applicable<br>Not recorded	
156	Q29.1	Have you seen in last 30 d info about tobacco	I	1	1<br>2<br>8	Yes<br>No<br>Not applicable	

		promotion/encourage - Television			9	Not recorded	
157	Q29.2	Have you seen in last 30 d info about tobacco promotion/encourage - Radio	I	1	1<br>2<br>8<br>9	Yes<br>No<br>Not applicable<br>Not recorded	
158	Q29.3	Have you seen in last 30 d info about tobacco promotion/encourage - Newspapers	I	1	1<br>2<br>8<br>9	Yes<br>No<br>Not applicable<br>Not recorded	
159	Q29.4	Have you seen in last 30 d info about tobacco promotion/encourage - Billiboards	I	1	1<br>2<br>8<br>9	Yes<br>No<br>Not applicable<br>Not recorded	
160	Q29.5	Have you seen in last 30 d info about tobacco promotion/encourage -Tobacco packs	I	1	1<br>2<br>8<br>9	Yes<br>No<br>Not applicable<br>Not recorded	
161	Q29.6	Have you seen in last 30 d info about tobacco promotion/encourage -Movies	I	1	1<br>2<br>8<br>9	Yes<br>No<br>Not applicable<br>Not recorded	
162	Q29.7	Have you seen in last 30 d info about tobacco promotion/encourage -Theatres	I	1	1<br>2<br>8<br>9	Yes<br>No<br>Not applicable<br>Not recorded	
163	Q29.8	Have you seen in last 30 d info about tobacco promotion/encourage -Not seen	I	1	1<br>2<br>8<br>9	Yes<br>No<br>Not applicable<br>Not recorded	
164	Q30	Do you suggest any measures for curbing the use of tobacco	S	50			Mention “missing” if no suggestion given

4. HHTN.CHK

```
LABELBLOCK
  LABEL label_q3a
    1  yes
    2  no
  END
END

BEFORE FILE
  HELP "Welcome data entry operator, Please do not use MOUSE for data entry. Use enter / tab /
arrow key to move from one field to another"
  DEFINE StartTime #####.######
END

AFTER FILE
  HELP "Thank you very much for your time"
END

BEFORE RECORD
  StartTime=NOW
END

AFTER RECORD
  IF idhouse=. THEN
    HELP "idhouse is unique identifier. You cannot proceed unless it is generated"
    GOTO distarea
  ENDIF
  IF secs=. THEN
    secs=(NOW-StartTime)*86400
  ELSE
    secs=secs+(NOW-StartTime)*86400
  ENDIF
END

secs
  NOENTER
END

idhouse
  KEY UNIQUE 1
  NOENTER
END

distcode
  NOENTER
END

distcopy
  NOENTER
```

4. HHTN.CHK

```
END

pass
  NOENTER
END

distarea
  COMMENT LEGAL site.rec SHOW
  MUSTENTER
  REPEAT
  TYPE COMMENT distcode
  AFTER ENTRY
    DISTCOPY=DISTAREA
  END
END

hhno
  RANGE 1 2999
  MUSTENTER
  AFTER ENTRY
    idhouse=distcode+"-"+hhno
  END
END

q1
  RANGE 1 20
  LEGAL
    99
  END
  MUSTENTER
END

q2
  RANGE 1 20
  LEGAL
    99
  END
  MUSTENTER
  AFTER ENTRY
    IF q2>q1 THEN
      HELP "Number of persons >=15 years cannot be greater than total people, Please verify"
      GOTO q1
    ENDIF
  END
END

q2a
  RANGE 0 20
```

4. HHTN.CHK

```
  LEGAL
    99
  END
  MUSTENTER
END

q2b
  RANGE 0 20
  LEGAL
    99
  END
  MUSTENTER
  AFTER ENTRY
    IF q2a+q2b<>q2 THEN
      HELP "The sum of males and females is not matching the total"
      GOTO q2
    ENDIF
  END
END

q3a
  COMMENT LEGAL USE label_q3a SHOW
  MUSTENTER
  TYPE COMMENT
END

q3b
  COMMENT LEGAL USE label_q3a SHOW
  MUSTENTER
  TYPE COMMENT
END

q3c
  COMMENT LEGAL USE label_q3a SHOW
  MUSTENTER
  TYPE COMMENT
END

q3d
  COMMENT LEGAL USE label_q3a SHOW
  MUSTENTER
  TYPE COMMENT
END

q3e
  COMMENT LEGAL USE label_q3a SHOW
  MUSTENTER
  TYPE COMMENT
```

4. HHTN.CHK

```
END

q3f
  COMMENT LEGAL USE label_q3a SHOW
  MUSTENTER
  TYPE COMMENT
END

q3g
  COMMENT LEGAL USE label_q3a SHOW
  MUSTENTER
  TYPE COMMENT
END

q3h
  COMMENT LEGAL USE label_q3a SHOW
  MUSTENTER
  TYPE COMMENT
END

q3i
  COMMENT LEGAL USE label_q3a SHOW
  MUSTENTER
  TYPE COMMENT
END

q3j
  COMMENT LEGAL USE label_q3a SHOW
  MUSTENTER
  TYPE COMMENT
END

deo
  MUSTENTER
  REPEAT
END
```

5. HHTN.QES

```
Study name: Tamil Nadu Tobacco Survey
Dear Data entry operator, Do not use mouse or key pad during data entry.
Use enter/tab/arrow key(s) to move from one field to another
Press F9 if options do not appear for categorical variable
Keep a printout of data documentation sheet by your side during data
entry
Contact Mr Prabhakar in case of any query during data entry 99622 76628

secs                       Time in seconds to fill this record ####
noenter
idhouse                             Unique id for the household ___________
unique id, noenter field
distcode                                          district code ______
noenter field
distcopy                                    Copy of the distarea
________________________________________ noenter field
pass                                                       code <E       >

distarea                   name of the district including area
________________________________________
hhno                                      household serial number #### enter
2001, 2002, 2003 onwards if missing/non unique, mention the same in
questionnaire, press F5 to make a note

Q1                         number of household members/persons ## enter 99
if the data is not recorded
Q2              number of household members of age >/= 15 yrs ## enter 99
if the data is not recorded
Q2a        number of household male members of age >/= 15 yrs ## enter 99
if the data is not recorded
Q2b      number of household female members of age >/= 15 yrs ## enter 99
if the data is not recorded

Q3a                                                 electricity #
Q3b                                                flush toilet #
Q3c                                            fixed telephone #
Q3d                                                      mobile #
Q3e                                                  television #
Q3f                                                       radio #
Q3g                                                refrigerator #
Q3h                                                         car #
Q3i                                                 two wheeler #
Q3j                                             washing machine #
deo                                   name of dataentry operator
____________________
```

6. HHTN.REC

```
38 1 VLAB ~kq:CM89s2jGP9H=:kq~ Filelabel: TNTS-HOUSEHOLD
_label1        1   1  30   0   0   0   0 112 Study name: Tamil Nadu
Tobacco Survey
_label2        1   2  30   0   0   0   0 112 Dear Data entry operator, Do
not use mouse or key pad during data entry.
_label3        1   3  30   0   0   0   0 112 Use enter/tab/arrow key(s)
to move from one field to another
_label4        1   4  30   0   0   0   0 112 Press F9 if options do not
appear for categorical variable
_label5        1   5  30   0   0   0   0 112 Keep a printout of data
documentation sheet by your side during data entry
_label6        1   6  30   0   0   0   0 112 Contact Mr Prabhakar in case
of any query during data entry 99622 76628
#secs          1   8  30  63   8   0   4 112 secs
Time in seconds to fill this record
_label7       67   8  30   0   0   0   0 112  noenter
_idhouse       1   9  30  63   9   1  11 112 idhouse
Unique id for the household
_label8       74   9  30   0   0   0   0 112  unique id, noenter field
_distcode      1  10  30  63  10   1   6 112 distcode
district code
_label9       69  10  30   0   0   0   0 112  noenter field
_distcopy      1  11  30  63  11   1  40 112 distcopy
Copy of the distarea
_label10     103  11  30   0   0   0   0 112  noenter field
_pass          1  12  30  63  12  18  12 119 pass
code
_distarea      1  14  30  63  14   1  40 112 distarea
name of the district including area
#hhno          1  15  30  63  15   0   4 112 hhno
household serial number
_label11      67  15  30   0   0   0   0 112  enter 2001, 2002, 2003
onwards if missing/non unique, mention the same in quest
_label12     147  15  30   0   0   0   0 112 ionnaire, press F5 to make a
note
#q1            1  17  30  63  17   0   2 112 q1
number of household members/persons
_label13      65  17  30   0   0   0   0 112  enter 99 if the data is not
recorded
#q2            1  18  30  63  18   0   2 112 q2              number of
household members of age >/= 15 yrs
_label14      65  18  30   0   0   0   0 112  enter 99 if the data is not
recorded
#q2a           1  19  30  63  19   0   2 112 q2a        number of
household male members of age >/= 15 yrs
_label15      65  19  30   0   0   0   0 112  enter 99 if the data is not
recorded
#q2b           1  20  30  63  20   0   2 112 q2b      number of household
female members of age >/= 15 yrs
_label16      65  20  30   0   0   0   0 112  enter 99 if the data is not
recorded
#q3a           1  22  30  63  22   0   1 112 q3a
electricity
#q3b           1  23  30  63  23   0   1 112 q3b
flush toilet
#q3c           1  24  30  63  24   0   1 112 q3c
fixed telephone
```

6. HHTN.REC

```
#q3d           1  25  30  63  25   0   1 112 q3d
mobile
#q3e           1  26  30  63  26   0   1 112 q3e
television
#q3f           1  27  30  63  27   0   1 112 q3f
radio
#q3g           1  28  30  63  28   0   1 112 q3g
refrigerator
#q3h           1  29  30  63  29   0   1 112 q3h
car
#q3i           1  30  30  63  30   0   1 112 q3i
two wheeler
#q3j           1  31  30  63  31   0   1 112 q3j
washing machine
_deo           1  32  30  63  32   1  20 112 deo
name of dataentry operator
```

7. INDITN.QES

Study name: Tamil Nadu Tobacco Survey
Dear Data entry operator, Do not use mouse or key pad during data entry.
Use enter/tab/arrow key(s) to move from one field to another
Press F9 if options do not appear for categorical variable
Keep a printout of data documentation sheet by your side during data entry
Contact Mr Prabhakar in case of any query during data entry 99622 76628

secs Time in seconds to fill this record #### noenter
idindi unique id for individual __________________ unique id, no enter field
idhouse Unique id for the household ___________ key id, noenter field
distcode
district code ______ noenter field
distcopy Copy of the distarea ____ noenter field
pass
code <E >

deo name of dataentry operator ____________________
distarea name of the district including area ____
hhno household serial number #### enter 2001, 2002, 2003 onwards if missing/non unique, mention the same in questionnaire, press F5 to make a note
indino individual participant id no __________ use appropriate id in case of missing esp those >15y olds who do not give interview

Individual Questionnaire
name
Participant's name ____
gender participant's gender #
age
participant's age ### enter 999 if the data is not recorded
occ participant's occupation #
edn participant's education #
phno phone number ########## enter 99999999999 (10 digit) for missing, do not enter 0 or +91 before the number

filled was questionnaire filled #
reason why was the questionnaire not filled #

Q1 Do you currently use tobacco? #
Q2 In the past, have you used tobacco? #

Smoking and Chewing Habits

7. INDITN.QES

Q3sma whether user of tobacco smoking Cigarettes (filtered/non-filtered) # mention yes if row in Q3 table is filled. This variable is only for efficient data entry, not to be used/included in analysis
Q3sma1 name of the tobacco smoking Cigarettes (filtered/non-filtered) __________________ mention not applicable if required
Q3sma2 cigarettes (filtered/non-filtered) smoking Current/Past #
Q3sma3d cigarettes (filtered/non-filtered)daily frequency ## enter 99 if the data is not recorded, 98 if not applicable
Q3sma3w cigarettes (filtered/non-filtered)weekly frequency ### enter 999 if the data is not recorded, 998 if not applicable
Q3sma4 cigarettes (filtered/non-filtered) age started in ## enter 99 if the data is not recorded, 98 if not applicable
Q3sma5 cigarettes (filtered/non-filtered) age past user stopped at ### enter 999 if the data is not recorded, 998 if not applicable
Q3sma6 cigarettes (filtered/non-filtered) duration of use ## noenter

Q3smb whether user of tobacco smoking bidi # mention yes if row in Q3 table is filled. This variable is only for efficient data entry, not to be used/included in analysis
Q3smb1 name of the tobacco smoking bidi brand __________________ mention not applicable if required
Q3smb2 bidi smoking Current/Past #
Q3smb3d bidi smoking daily frequency ## enter 99 if the data is not recorded, 98 if not applicable
Q3smb3w bidi smoking weekly frequency ### enter 999 if the data is not recorded, 998 if not applicable
Q3smb4 bidi smoking age started in ## enter 99 if the data is not recorded, 98 if not applicable
Q3smb5 bidi smoking age past user stopped at ### enter 999 if the data is not recorded, 998 if not applicable
Q3smb6 bidi smoking duration of use ## noenter

Q3smc whether user of tobacco smoking Chutta/Cigar/Cheroot # mention yes if row in Q3 table is filled. This variable is only for efficient data entry, not to be used/included in analysis
Q3smc1 name of the tobacco smoking Chutta/Cigar/Cheroot brand __________________ mention not applicable if required
Q3smc2 Chutta/Cigar/Cheroot smoking Current/Past #
Q3smc3d Chutta/Cigar/Cheroot smoking daily frequency ## enter 99 if the data is not recorded, 98 if not applicable
Q3smc3w Chutta/Cigar/Cheroot smoking weekly frequency ### enter 999 if the data is not recorded, 998 if not applicable
Q3smc4 Chutta/Cigar/Cheroot smoking age started in ## enter 99 if the data is not recorded, 98 if not applicable
Q3smc5 Chutta/Cigar/Cheroot smoking age past user stopped at ### enter 999 if the data is not recorded, 998 if not applicable

Q3smc6 Chutta/Cigar/Cheroot smoking duration of use ## noenter

Q3smd whether user of tobacco smoking others # mention yes if row in Q3 table is filled. This variable is only for efficient data entry, not to be used/included in analysis
Q3smd1 name of the tobacco smoking Others brand __________________ mention not applicable if required
Q3smd2 Others smoking Current/Past #
Q3smd3d Others smoking daily frequency ## enter 99 if the data is not recorded, 98 if not applicable
Q3smd3w Others smoking weekly frequency ### enter 999 if the data is not recorded, 998 if not applicable
Q3smd4 Others smoking age started in ## enter 99 if the data is not recorded, 98 if not applicable
Q3smd5 Others smoking age past user stopped at ### enter 999 if the data is not recorded, 998 if not applicable
Q3smd6 Others smoking duration of use ## noenter

Q3cha whether user of tobacco Chewing alone # mention yes if row in Q3 table is filled. This variable is only for efficient data entry, not to be used/included in analysis
Q3cha1 name of the chewing tobacco alone __________________ mention not applicable if required
Q3cha2 tobacco alone chewing Current/Past #
Q3cha3d tobacco alone chewing daily frequency ## enter 99 if the data is not recorded, 98 if not applicable
Q3cha3w tobacco alone chewing weekly frequency ### enter 999 if the data is not recorded, 998 if not applicable
Q3cha4 tobacco alone chewing age started in ## enter 99 if the data is not recorded, 98 if not applicable
Q3cha5 tobacco alone chewing past user stopped at ### enter 999 if the data is not recorded, 998 if not applicable
Q3cha6 tobacco alone chewing duration of use ## noenter

Q3chb whether user of chewing TOBACCO/AN/PAN # mention yes if row in Q3 table is filled. This variable is only for efficient data entry, not to be used/included in analysis
Q3chb1 name of the chewing TOBACCO/AN/PAN __________________ mention not applicable if required
Q3chb2 TOBACCO/AN/PAN chewing Current/Past #
Q3chb3d TOBACCO/AN/PAN chewing daily frequency ## enter 99 if the data is not recorded, 98 if not applicable
Q3chb3w TOBACCO/AN/PAN chewing weekly frequency ### enter 999 if the data is not recorded, 998 if not applicable
Q3chb4 TOBACCO/AN/PAN chewing age started in ## enter 99 if the data is not recorded, 98 if not applicable

7. INDITN.QES

Q3chb5 TOBACCO/AN/PAN chewing past user stopped at ### enter 999 if the data is not recorded, 998 if not applicable
Q3chb6 TOBACCO/AN/PAN chewing duration of use ## noenter

Q3chc whether user of chewing Tobacco+Pan # mention yes if row in Q3 table is filled. This variable is only for efficient data entry, not to be used/included in analysis
Q3chc1 name of the chewing Tobacco+Pan __________________ mention not applicable if required
Q3chc2 Tobacco+Pan chewing Current/Past #
Q3chc3d Tobacco+Pan chewing daily frequency ## enter 99 if the data is not recorded, 98 if not applicable
Q3chc3w Tobacco+Pan chewing weekly frequency ### enter 999 if the data is not recorded, 998 if not applicable
Q3chc4 Tobacco+Pan chewing age started in ## enter 99 if the data is not recorded, 98 if not applicable
Q3chc5 Tobacco+Pan chewing past user stopped at ### enter 999 if the data is not recorded, 998 if not applicable
Q3chc6 Tobacco+Pan duration of use ## noenter

Q3chd whether user of chewing tobacco,others # mention yes if row in Q3 table is filled. This variable is only for efficient data entry, not to be used/included in analysis
Q3chd1 name of the chewing tobacco,others __________________ mention not applicable if required
Q3chd2 tobacco,others chewing Current/Past #
Q3chd3d tobacco,others chewing daily frequency ## enter 99 if the data is not recorded, 98 if not applicable
Q3chd3w tobacco,others chewing weekly frequency ### enter 999 if the data is not recorded, 998 if not applicable
Q3chd4 tobacco,others chewing age started in ## enter 99 if the data is not recorded, 98 if not applicable
Q3chd5 tobacco,others chewing past user stopped at ### enter 999 if the data is not recorded, 998 if not applicable
Q3chd6 tobacco,others chewing duration of use ## noenter

Q3sna whether user of snuffing tobacco, by mouth # mention yes if row in Q3 table is filled. This variable is only for efficient data entry, not to be used/included in analysis
Q3sna1 name of the snuffing tobacco, by mouth __________________ mention not applicable if required
Q3sna2 snuffing by mouth Current/Past #
Q3sna3d snuffing by mouth daily frequency ## enter 99 if the data is not recorded, 98 if not applicable
Q3sna3w snuffing by mouth weekly frequency ### enter 999 if the data is not recorded, 998 if not applicable

Q3sna4 snuffing by mouth age started in ## enter 99 if the data is not recorded, 98 if not applicable
Q3sna5 snuffing by mouth past user stopped at ### enter 999 if the data is not recorded, 998 if not applicable
Q3sna6 snuffing by mouth duration of use ## noenter

Q3snb whether user of snuffing tobacco, by nose # mention yes if row in Q3 table is filled. This variable is only for efficient data entry, not to be used/included in analysis
Q3snb1 name of the snuffing tobacco, by nose __________________ mention not applicable if required
Q3snb2 snuffing by nose Current/Past #
Q3snb3d snuffing by nose daily frequency ## enter 99 if the data is not recorded, 98 if not applicable
Q3snb3w snuffing by nose weekly frequency ### enter 999 if the data is not recorded, 998 if not applicable
Q3snb4 snuffing by nose age started in ## enter 99 if the data is not recorded, 98 if not applicable
Q3snb5 snuffing by nose past user stopped at ### enter 999 if the data is not recorded, 998 if not applicable
Q3snb6 snuffing by nose duration of use ## noenter

Q3ala whether user of alcohol # mention yes if row in Q3 table is filled. This variable is only for efficient data entry, not to be used/included in analysis
Q3ala1 name of the alcohol use __________________ mention not applicable if required
Q3ala2 alcohol Current/Past #
Q3ala3d alcohol daily frequency ## enter 99 if the data is not recorded, 98 if not applicable
Q3ala3w alcohol weekly frequency ### enter 999 if the data is not recorded, 998 if not applicable
Q3ala4 alcohol age started in ## enter 99 if the data is not recorded, 98 if not applicable
Q3ala5 alcohol past user stopped at ### enter 999 if the data is not recorded, 998 if not applicable
Q3ala6 alcohol duration of use ## noenter

Q4 post wake up, when do you usually have your first smoke/chew/snuff? #
Q5 number of cigarettes/Bidis/Packets purchased last time ##.# 99.9 not recorded, 99.8 not applicable
Q5a If it is a pack, how many sticks were there in the packet? ## 99 not recorded, 98 not applicable
Q6 In total, how much money did you pay for this purchase (in INR)? ####.## 9999.99 not recorded, 9999.98 not applicable
Q7 total expense on this purchase (in INR) till date #########.## 9999.99 not recorded, 9999.98 not applicable

7. INDITN.QES

```
Q8                                    Are you concerned about your
tobacco use? #
Q9                             Have you visited a doctor in the past 12
months? #
Q10                     Did the doctor ask if you use Tobacco (in last
12mths)? #
Q11                      Did the doctor advise to quit Tobacco(in last
12mths)? #
Q12          Inlast 30days,didyou notice any warning on tobacco product
youuse? #
Q13          In last 30days,did warning labels led you to think about
quitting? #
Q14* In past 12 months, which method did you try to stop using tobacco?
Q148                                                                 no
attempt #
Q141
counselling #
Q142                                                nicotine replacement
therapy #
Q143
medications #
Q144                                              switching to alternate
tobacco #
Q145                                                               a
quit line #
Q146                                                       traditional
medicines #
Q147                                                            quit on
my own #
Q149                                                        other method
to quit #
Q15           Ifyou have quit completely,specify the method you found
effective #
Q16                       How long did you stop using tobacco, since last
quit? #
Q17               Which of the following best describes about quitting
tobacco? #
If less than 18 years
Q18              Were you refused while purchasing tobacco in the past 30
days? #
If Chewers
Q19               Did you find it difficult to purchase tobacco in past
30days? #
Q20                           Brand name of the tobacco that is usually
purchased ____________________ mention not applicable if required
Q20a                Price of the tobacco brand (usually purchased)before
the ban ####.## 9999.99 not recorded, 9999.98 not applicable
Q20b                 Price of the tobacco brand (usually purchased)after
the ban ####.## 9999.99 not recorded, 9999.98 not applicable

For Non-users as well as users of tobacco in the household

Q21                           Do you know the harmful effects of passive
smoking? #
Q22                    Which describes the best rule for smoking inside your
home? #
Q23a                                              Does anyone smoke
inside home #
```

7. INDITN.QES

Q23b How often does anyone smoke inside your home #
Q24 Which best describes the indoor smoking policy where you work? #
Q25 During past 30days,did anyone smoke in indoor areas where you work? #
Q26 Does breathing,others smoke cause serious illness in Non-smokers? #
Q27* Does tobacco cause the following (Based on what you know or believe)
Q271 stroke #
Q272 heart attack #
Q273 cancer #
Q274 cause serious illness #
Q275 infertility/impotence #
Q276 don't know #
Q28* In last 30 days,any information seen on tobacco danger or quitting?
Q288 not seen #
Q281 television #
Q282 radio #
Q283 newspapers #
Q284 billiboards #
Q285 tobacco packs #
Q286 movies #
Q287 theatres #
Q29* In last 30days,any advertisement was seen promoting tobacco products?
Q298 not seen #
Q291 television #
Q292 radio #
Q293 newspapers #
Q294 billiboards #
Q295 tobacco packs #
Q296 movies #
Q297 theatres #

7. INDITN.QES

Q30 Do you suggest any measures for curbing the use of tobacco? ____ mention "missing" if no suggestion

8. INDITN.CHK

```
LABELBLOCK
  LABEL label_gender    1  male    2  female    3  transgender    9  "not
recorded"  END
  LABEL label_occ    0  student    1  unemployed    2  homemaker    3
"Daily wage"    4  driver    5  "private job"    6  "Govt employed"    7
"Self employed"    8  Retired    9  "Not recorded"  END
  LABEL label_edn    1  "no formal schooling"    2  "primary (1st TO
5th)"    3  "Secondary (6th to 10th)"    4  "Higher Secondary (+1, +2)"
5  "Degree or higher"    9  "not recorded"  END
  LABEL label_filled    1  yes    2  no  END
  LABEL label_reason    1  "not available"    2  "did not consent"    8
"not applicable"    9  "not recorded"  END
  LABEL label_q1    1  yes    2  no    8  "not applicable"    9  "not
recorded"  END
  LABEL label_q2    1  yes    2  no    8  "not applicable"    9  "not
recorded"  END
  LABEL label_q3sma    1  yes    2  no  END
  LABEL label_q3sma1    Kings  1    Scissors  2    "Gold flake"  3
Flake  4    Lites  5    Charminar  6    Wills  7    Charms  8    Panama
9    Black  10    "Wills classic"  11    Cavanders  12    555  13
Total  14    Marlboro  15    Editions  16    Classic  17    Camel  8
Lites  19    B&H  20    Others  21    Missing  99  END
  LABEL label_q3sma2    1  current    2  past    8  "not applicable"    9
"not recorded"  END
  LABEL label_q3smb1    Seyadu  1    Ganesh  2    Chandrika  3    Kajah
4    Arasan  5    "Ball Biri"  6    GT  7    JD  8    Kailash  9    "Om
Shankar"  10    Others  11    Missing  99  END
  LABEL label_q3smb2    1  current    2  past    8  "not applicable"    9
"not recorded"  END
  LABEL label_q3cha1    Hans  1    MDM  2    MAWA  3    "Cool lip"  4
"Chaini Khaini"  5    Garuda  6    Swagat  7    Rajnigandha  8    MIRAJ
9    M  10    "Super shanthi"  11    RMD  12    Others  13    Missing  99
END
  LABEL label_q3sna1    Uma  1    DS  2    Others  3    Missing  9  END
  LABEL label_q3snb2    1  current    2  past    8  "not applicable"    9
"not recorded"  END
  LABEL label_q4    1  "within 5 minutes"    2  "6-30 minutes"    3  "31-
60 minutes"    4  "more than 60 min"    8  "not applicable"    9  "not
recorded"  END
  LABEL label_q15    0  counseling    1  "nicotine replacement"    2
medications    3  "Switiching to aternate tobacco"    4  "a quit line"
5  "traditional medicines"    6  "quit on my own"    7  other    8  "not
applicable"    9  "not recorded"  END
  LABEL label_q16    1  months    2  weeks4    3  days    4  "less than 1
day"    5  "no change"    8  "not applicable"    9  "not recorded"  END
  LABEL label_q17    1  "within 1 month"    2  "within 12 months"    3
"quit someday, but not next 12 mo"    4  "Dont know"    5  "not
interested in quiting"    8  "not applicable"    9  "not recorded"  END
  LABEL label_q22    1  allowed    2  "not allowed, exceptions"    3
"never allowed"    4  "no rules"    5  "Don't know"    8  "not applicable
"    9  "not recorded"  END
  LABEL label_q23b    1  daily    2  weekly    3  <weekly    4  <monthly
5  never    6  "dont know"    8  "not applicable"    9  "not recorded"
END
  LABEL label_q24    1  "allowed anywhere"    2  "allowed in some indoor
areas"    3  "only in outdoor areas"    4  "not allowed"    5  "there is
not policy"    6  "dont know"    8  "not applicable"    9  "not recorded"
END
```

8. INDITN.CHK

```
  LABEL label_q25    1  yes    2  no    3  "dont know"    8  "not
applicable"    9  "not recorded"  END
  LABEL label_q30    1  "Ban tobacco sale / production"    2  Awareness
3  "quit tobacco"    4  "stringent enforcement of laws/pu"    5  "Bring
alternative to tobacco"    6  "no comments"    7  others  END
END

BEFORE FILE
  DEFINE StartTime #####.#####
  HELP "Dear data entry operator, Do not use mouse. Use enter/tab/arrow
key to move from one field to another"
  HELP "Dear Data entry operator, please ensure you have a hard copy of
data documentation sheet before you start data entry. Also, do not use
mouse for data entry"
END

AFTER FILE
  HELP "Thank you for data entry"
  HELP "Thank you very much for data entry. We appreciate the hard work
from your end"
END

BEFORE RECORD
  StartTime=NOW
END

AFTER RECORD
  IF idindi=. THEN
    HELP "cannot proceed without idindi. It is unique id"
    GOTO distarea
  ENDIF
  IF idhouse=. THEN
    HELP "cannot proceed without idhouse. It is key id"
    GOTO distarea
  ENDIF
  IF secs=. THEN
    secs=(NOW-StartTime)*86400
  ELSE
    secs=secs+(NOW-StartTime)*86400
  ENDIF
  IF q3sma6<0 THEN
    HELP "q3sma6 cannot be less than zero. Please check q3sma4 and
q3sma5"
    GOTO q3sma4
  ENDIF
  IF q3smb6<0 THEN
    HELP "q3smb6 cannot be less than zero. Please check q3smb4 and
q3smb5"
    GOTO q3smb4
  ENDIF
  IF q3smc6<0 THEN
    HELP "q3smc6 cannot be less than zero. Please check q3smc4 and
q3smc5"
    GOTO q3smc4
  ENDIF
  IF q3smd6<0 THEN
    HELP "q3smd6 cannot be less than zero. Please check q3smd4 and
q3smd5"
```

8. INDITN.CHK

```
    GOTO q3smd4
  ENDIF
  IF q3cha6<0 THEN
    HELP "q3cha6 cannot be less than zero. Please check q3cha4 and
q3cha5"
    GOTO q3cha4
  ENDIF
  IF q3chb6<0 THEN
    HELP "q3chb6 cannot be less than zero. Please check q3chb4 and
q3chb5"
    GOTO q3chb4
  ENDIF
  IF q3chc6<0 THEN
    HELP "q3chc6 cannot be less than zero. Please check q3chc4 and
q3chc5"
    GOTO q3cha4
  ENDIF
  IF q3chd6<0 THEN
    HELP "q3chd6 cannot be less than zero. Please check q3chd4 and
q3chd5"
    GOTO q3chd4
  ENDIF
  IF q3sna6<0 THEN
    HELP "q3sna6 cannot be less than zero. Please check q3sna4 and
q3sna5"
    GOTO q3sna4
  ENDIF
  IF q3snb6<0 THEN
    HELP "q3snb6 cannot be less than zero. Please check q3snb4 and
q3snb5"
    GOTO q3snb4
  ENDIF
  IF q3ala6<0 THEN
    HELP "q3ala6 cannot be less than zero. Please check q3ala4 and
q3ala5"
    GOTO q3ala4
  ENDIF
  IF idindi=. THEN
    HELP "You cannot proceed without idindi generated" TYPE=WARNING
    GOTO distarea
  ENDIF
  IF idhouse=. THEN
    HELP "You cannot proceed without idhouse generated" TYPE=WARNING
    GOTO distarea
  ENDIF
END

secs
  NOENTER
END

idindi
  KEY UNIQUE 1
  NOENTER
END

idhouse
  KEY 2
```

8. INDITN.CHK

```
  NOENTER
END

distcode
  NOENTER
END

distcopy
  NOENTER
END

pass
  NOENTER
END

deo
  MUSTENTER
  REPEAT
END

distarea
  COMMENT LEGAL site.rec SHOW
  MUSTENTER
  REPEAT
  TYPE COMMENT distcode
  AFTER ENTRY
    DISTCOPY=DISTAREA
  END
END

hhno
  RANGE 1 2999
  MUSTENTER
  AFTER ENTRY
    idhouse=distcode+"-"+hhno
  END
END

indino
  MUSTENTER
  AFTER ENTRY
    idindi=distcode+"-"+HHNO+"-"+indino
  END
END

name
  MUSTENTER
END

gender
  COMMENT LEGAL USE label_gender SHOW
  MUSTENTER
  TYPE COMMENT
END

age
  RANGE 15 110
  LEGAL
```

```
    999
  END
  MUSTENTER
  AFTER ENTRY
    IF age>17 THEN
      q18=8
      GOTO occ
    ENDIF
  END
END

occ
  COMMENT LEGAL USE label_occ SHOW
  MUSTENTER
  TYPE COMMENT
  AFTER ENTRY
    IF occ<3 THEN
      q24=8
      Q25=8
      GOTO edn
    ENDIF
    IF occ>7 THEN
      q24=8
      Q25=8
      GOTO edn
    ENDIF
  END
END

edn
  COMMENT LEGAL USE label_edn SHOW
  MUSTENTER
  TYPE COMMENT
END

phno
  RANGE 6000000000 9999999999
  MUSTENTER
END

filled
  COMMENT LEGAL USE label_filled SHOW
  MUSTENTER
  TYPE COMMENT
  AFTER ENTRY
    IF filled=1 THEN
      reason=8
      GOTO q1
    ENDIF
  END
END

reason
  COMMENT LEGAL USE label_reason SHOW
  MUSTENTER
  TYPE COMMENT
  AFTER ENTRY
    IF (reason=8) and (filled=2) THEN
```

```
    HELP "reason and filled not compatible. Please recheck"
    GOTO filled
  ENDIF
  IF filled=2 THEN
    q1=8
    q2=8
    q3sma=8
    q3sma1="not applicable"
    q3sma2=8
    q3sma3d=98
    q3sma3w=998
    q3sma4=98
    q3sma5=998
    q3sma6=98
    q3smb=8
    q3smb1="not applicable"
    q3smb2=8
    q3smb3d=98
    q3smb3w=998
    q3smb4=98
    q3smb5=998
    q3smb6=98
    q3smc=8
    q3smc1="not applicable"
    q3smc2=8
    q3smc3d=98
    q3smc3w=998
    q3smc4=98
    q3smc5=998
    q3smc6=98
    q3smd=8
    q3smd1="not applicable"
    q3smd2=8
    q3smd3d=98
    q3smd3w=998
    q3smd4=98
    q3smd5=998
    q3smd6=98
    q3cha=8
    q3cha1="not applicable"
    q3cha2=8
    q3cha3d=98
    q3cha3w=998
    q3cha4=98
    q3cha5=998
    q3cha6=98
    q3chb=8
    q3chb1="not applicable"
    q3chb2=8
    q3chb3d=98
    q3chb3w=998
    q3chb4=98
    q3chb5=998
    q3chb6=98
    q3chc=8
    q3chc1="not applicable"
    q3chc2=8
    q3chc3d=98
```

8. INDITN.CHK

```
q3chc3w=998
q3chc4=98
q3chc5=998
q3chc6=998
q3chd=8
q3chd1="not applicable"
q3chd2=8
q3chd3d=98
q3chd3w=998
q3chd4=98
q3chd5=998
q3chd6=98
q3sna=8
q3sna1="not applicable"
q3sna2=8
q3sna3d=98
q3sna3w=998
q3sna4=98
q3sna5=998
q3sna6=98
q3snb=8
q3snb1="not applicable"
q3snb2=8
q3snb3d=98
q3snb3w=998
q3snb4=98
q3snb5=998
q3snb6=98
q3ala=8
q3ala1="not applicable"
q3ala2=8
q3ala3d=98
q3ala3w=998
q3ala4=98
q3ala5=998
q3ala6=98
q4=8
q5=98
q5a=98
q6=9999.98
q7=9999.98
q8=8
q9=8
q10=8
q11=8
q12=8
q13=8
q141=8
q142=8
q143=8
q144=8
q145=8
q146=8
q147=8
q148=8
q149=8
q15=8
q16=8
```

```
      q17=8
      q18=8
      q19=8
      q20="not applicable"
      q20a=9999.98
      q20b=9999.98
      q21=8
      q22=8
      q23a=8
      q23b=8
      q24=8
      q25=8
      q26=8
      q271=8
      q272=8
      q273=8
      q274=8
      q275=8
      q276=8
      q281=8
      q282=8
      q283=8
      q284=8
      q285=8
      q286=8
      q287=8
      q288=8
      q291=8
      q292=8
      q293=8
      q294=8
      q295=8
      q296=8
      q297=8
      q298=8
      q30="not applicable"
      GOTO WRITE
    ENDIF
  END
END

q1
  COMMENT LEGAL USE label_q1 SHOW
  MUSTENTER
  TYPE COMMENT
  AFTER ENTRY
    IF q1=1 THEN
      q2=8
      q3sma5=998
      q3smb5=998
      q3smc5=998
      q3smd5=998
      q3cha5=998
      q3chb5=998
      q3chc5=998
      q3chd5=998
      q3sna5=998
      q3snb5=998
```

8. INDITN.CHK

```
      GOTO q3sma
    ENDIF
  END
END

q2
  COMMENT LEGAL USE label_q2 SHOW
  MUSTENTER
  TYPE COMMENT
  AFTER ENTRY
    IF (q1=2) and (q2=2) THEN
      q3sma=8
      q3sma1="not applicable"
      q3sma2=8
      q3sma3d=98
      q3sma3w=998
      q3sma4=98
      q3sma5=998
      q3sma6=98
      q3smb=8
      q3smb1="not applicable"
      q3smb2=8
      q3smb3d=98
      q3smb3w=998
      q3smb4=98
      q3smb5=998
      q3smb6=98
      q3smc=8
      q3smc1="not applicable"
      q3smc2=8
      q3smc3d=98
      q3smc3w=998
      q3smc4=98
      q3smc5=998
      q3smc6=98
      q3smd=8
      q3smd1="not applicable"
      q3smd2=8
      q3smd3d=98
      q3smd3w=998
      q3smd4=98
      q3smd5=998
      q3smd6=98
      q3cha=8
      q3cha1="not applicable"
      q3cha2=8
      q3cha3d=98
      q3cha3w=998
      q3cha4=98
      q3cha5=998
      q3cha6=98
      q3chb=8
      q3chb1="not applicable"
      q3chb2=8
      q3chb3d=98
      q3chb3w=998
      q3chb4=98
      q3chb5=998
```

8. INDITN.CHK

```
q3chb6=98
q3chc=8
q3chc1="not applicable"
q3chc2=8
q3chc3d=98
q3chc3w=998
q3chc4=98
q3chc5=998
q3chc6=998
q3chd=8
q3chd1="not applicable"
q3chd2=8
q3chd3d=98
q3chd3w=998
q3chd4=98
q3chd5=998
q3chd6=98
q3sna=8
q3sna1="not applicable"
q3sna2=8
q3sna3d=98
q3sna3w=998
q3sna4=98
q3sna5=998
q3sna6=98
q3snb=8
q3snb1="not applicable"
q3snb2=8
q3snb3d=98
q3snb3w=998
q3snb4=98
q3snb5=998
q3snb6=98
q3ala=8
q3ala1="not applicable"
q3ala2=8
q3ala3d=98
q3ala3w=998
q3ala4=98
q3ala5=998
q3ala6=98
q4=8
q5=98
q5a=98
q6=9999.98
q7=9999.98
q8=8
q9=8
q10=8
q11=8
q12=8
q13=8
q141=8
q142=8
q143=8
q144=8
q145=8
q146=8
```

```
      q147=8
      q148=8
      q149=8
      q15=8
      q16=8
      q17=8
      q18=8
      q19=8
      q20="not applicable"
      q20a=9999.98
      q20b=9999.98
      GOTO q21
    ENDIF
    IF q2=1 THEN
      q4=8
      q5=98
      q5a=98
      q6=9999.98
      q7=9999.98
      q8=8
      q9=8
      q10=8
      q11=8
      q12=8
      q13=8
      q141=8
      q142=8
      q143=8
      q144=8
      q145=8
      q146=8
      q147=8
      q148=8
      q149=8
      q15=8
      q16=8
      q17=8
      q18=8
      q19=8
      q20="not applicable"
      q20a=9999.98
      q20b=9999.98
      GOTO q3sma
    ENDIF
  END
END

q3sma
  COMMENT LEGAL USE label_q3sma SHOW
  MUSTENTER
  AFTER ENTRY
    IF q3sma=2 THEN
      q3sma1="not applicable"
      q3sma2=8
      q3sma3d=98
      q3sma3w=998
      q3sma4=98
      q3sma5=998
```

```
      q3sma6=98
      GOTO q3smb
    ENDIF
  END
END

q3sma1
  COMMENT LEGAL USE label_q3sma1 SHOW
  MUSTENTER
  TYPE COMMENT
END

q3sma2
  COMMENT LEGAL USE label_q3sma2 SHOW
  MUSTENTER
  TYPE COMMENT
END

q3sma3d
  RANGE 1 70
  LEGAL
    98
    99
  END
  MUSTENTER
  AFTER ENTRY
    IF q3sma3d<71 THEN
      q3sma3w=998
      GOTO q3sma4
    ENDIF
  END
END

q3sma3w
  RANGE 1 490
  LEGAL
    998
    999
  END
  MUSTENTER
END

q3sma4
  RANGE 3 99
  MUSTENTER
END

q3sma5
  RANGE 3 110
  LEGAL
    998
    999
  END
  MUSTENTER
  AFTER ENTRY
    IF (q3sma5<>998) and (q3sma5>age) THEN
      HELP "q3sma5 cannot be more than age"
      CLEAR q3sma5
```

```
      GOTO q3sma5
    ENDIF
    IF (q3sma2=2)and (q3sma5<998) and (q3sma4<98) THEN
      q3sma6=q3sma5-q3sma4
    ENDIF
    IF (q3sma2=1)and (age<998) and (q3sma4<98) THEN
      q3sma6=age-q3sma4
    ENDIF
  END
END

q3sma6
  NOENTER
  RANGE 0 99
END

q3smb
  COMMENT LEGAL USE label_q3sma SHOW
  MUSTENTER
  AFTER ENTRY
    IF q3smb=2 THEN
      q3smb1="not applicable"
      q3smb2=8
      q3smb3d=98
      q3smb3w=998
      q3smb4=98
      q3smb5=998
      q3smb6=98
      GOTO q3smc
    ENDIF
  END
END

q3smb1
  COMMENT LEGAL USE label_q3smb1 SHOW
  MUSTENTER
  TYPE COMMENT
END

q3smb2
  COMMENT LEGAL USE label_q3smb2 SHOW
  MUSTENTER
  TYPE COMMENT
END

q3smb3d
  RANGE 1 70
  LEGAL
    98
    99
  END
  MUSTENTER
  AFTER ENTRY
    IF q3smb3d<71 THEN
      q3smb3w=998
      GOTO q3smb4
    ENDIF
  END
```

```
END

q3smb3w
  RANGE 1 490
  LEGAL
    998
    999
  END
  MUSTENTER
END

q3smb4
  RANGE 3 99
  MUSTENTER
END

q3smb5
  RANGE 3 110
  LEGAL
    998
    999
  END
  MUSTENTER
  AFTER ENTRY
    IF (q3smb5<>998) AND (q3smb5>age) THEN
      HELP "q3smb5 cannot be more than age"
      CLEAR q3smb5
      GOTO q3smb5
    ENDIF
    IF (q3smb2=2)and (q3smb5<998) and (q3smb4<98) THEN
      q3smb6=q3smb5-q3smb4
    ENDIF
    IF (q3smb2=1)and (age<998) and (q3smb4<98) THEN
      q3smb6=age-q3smb4
    ENDIF
  END
END

q3smb6
  NOENTER
  RANGE 0 99
END

q3smc
  COMMENT LEGAL USE label_q3sma SHOW
  MUSTENTER
  AFTER ENTRY
    IF q3smc=2 THEN
      q3smc1="not applicable"
      q3smc2=8
      q3smc3d=98
      q3smc3w=998
      q3smc4=98
      q3smc5=998
      q3smc6=98
      GOTO q3smd
    ENDIF
  END
```

8. INDITN.CHK

```
END

q3smc1
  MUSTENTER
END

q3smc2
  COMMENT LEGAL USE label_q3sma2 SHOW
  MUSTENTER
  TYPE COMMENT
END

q3smc3d
  RANGE 1 70
  LEGAL
    98
    99
  END
  MUSTENTER
  AFTER ENTRY
    IF q3smc3d<71 THEN
      q3smc3w=998
      GOTO q3smc4
    ENDIF
  END
END

q3smc3w
  RANGE 1 490
  LEGAL
    998
    999
  END
  MUSTENTER
END

q3smc4
  RANGE 3 99
  MUSTENTER
END

q3smc5
  RANGE 3 110
  LEGAL
    998
    999
  END
  MUSTENTER
  AFTER ENTRY
    IF (q3smc5<>998) AND (q3smc5>age) THEN
      HELP "q3smc5 cannot be more than age"
      CLEAR q3smc5
      GOTO q3smc5
    ENDIF
    IF (q3smc2=2) and (q3smc5<998) and (q3smc4<98) THEN
      q3smc6=q3smc5-q3smc4
    ENDIF
    IF (q3smc2=1)and (age<998) and (q3smc4<98) THEN
```

8. INDITN.CHK

```
      q3smc6=age-q3smc4
    ENDIF
  END
END

q3smc6
  NOENTER
  RANGE 0 99
END

q3smd
  COMMENT LEGAL USE label_q3sma SHOW
  MUSTENTER
  AFTER ENTRY
    IF q3smd=2 THEN
      q3smd1="not applicable"
      q3smd2=8
      q3smd3d=98
      q3smd3w=998
      q3smd4=98
      q3smd5=998
      q3smd6=98
      GOTO q3cha
    ENDIF
  END
END

q3smd1
  MUSTENTER
END

q3smd2
  COMMENT LEGAL USE label_q3sma2 SHOW
  MUSTENTER
  TYPE COMMENT
END

q3smd3d
  RANGE 1 70
  LEGAL
    98
    99
  END
  MUSTENTER
  AFTER ENTRY
    IF q3smd3d<71 THEN
      q3smd3w=998
      GOTO q3smd4
    ENDIF
  END
END

q3smd3w
  RANGE 1 490
  LEGAL
    998
    999
  END
```

```
  MUSTENTER
END

q3smd4
  RANGE 3 99
  MUSTENTER
END

q3smd5
  RANGE 3 110
  LEGAL
    998
    999
  END
  MUSTENTER
  AFTER ENTRY
    IF (q3smd5<>998) AND (q3smd5>age) THEN
      HELP "q3smd5 cannot be more than age"
      CLEAR q3smd5
      GOTO q3smd5
    ENDIF
    IF (q3smd2=2)and (q3smd5<998) and (q3smd4<98) THEN
      q3smd6=q3smd5-q3smd4
    ENDIF
    IF (q3smd2=1)and (age<998) and (q3smd4<98) THEN
      q3smd6=age-q3smd4
    ENDIF
  END
END

q3smd6
  NOENTER
  RANGE 0 99
END

q3cha
  COMMENT LEGAL USE label_q3sma SHOW
  MUSTENTER
  AFTER ENTRY
    IF q3cha=2 THEN
      q3cha1="not applicable"
      q3cha2=8
      q3cha3d=98
      q3cha3w=998
      q3cha4=98
      q3cha5=998
      q3cha6=98
      GOTO q3chb
    ENDIF
  END
END

q3cha1
  COMMENT LEGAL USE label_q3cha1 SHOW
  MUSTENTER
  TYPE COMMENT
END
```

8. INDITN.CHK

```
q3cha2
  COMMENT LEGAL USE label_q3sma2 SHOW
  MUSTENTER
  TYPE COMMENT
END

q3cha3d
  RANGE 1 70
  LEGAL
    98
    99
  END
  MUSTENTER
  AFTER ENTRY
    IF q3cha3d<71 THEN
      q3cha3w=998
      GOTO q3cha4
    ENDIF
  END
END

q3cha3w
  RANGE 1 490
  LEGAL
    998
    999
  END
  MUSTENTER
END

q3cha4
  RANGE 3 99
  MUSTENTER
END

q3cha5
  RANGE 3 110
  LEGAL
    998
    999
  END
  MUSTENTER
  AFTER ENTRY
    IF (q3cha5<>998) AND (q3cha5>age) THEN
      HELP "q3cha5 cannot be more than age"
      CLEAR q3cha5
      GOTO q3cha5
    ENDIF
    IF (q3cha2=2) AND (q3cha5<998) and (q3cha4<98) THEN
      q3cha6=q3cha5-q3cha4
    ENDIF
    IF (q3cha2=1)and (age<998) and (q3cha4<98) THEN
      q3cha6=age-q3cha4
    ENDIF
  END
END

q3cha6
```

8. INDITN.CHK

```
  NOENTER
  RANGE 0 99
END

q3chb
  COMMENT LEGAL USE label_q3sma SHOW
  MUSTENTER
  AFTER ENTRY
    IF q3chb=2 THEN
      q3chb1="not applicable"
      q3chb2=8
      q3chb3d=98
      q3chb3w=998
      q3chb4=98
      q3chb5=998
      q3chb6=98
      GOTO q3chc
    ENDIF
  END
END

q3chb1
  MUSTENTER
END

q3chb2
  COMMENT LEGAL USE label_q3sma2 SHOW
  MUSTENTER
  TYPE COMMENT
END

q3chb3d
  RANGE 1 70
  LEGAL
    98
    99
  END
  MUSTENTER
  AFTER ENTRY
    IF q3chb3d<71 THEN
      q3chb3w=998
      GOTO q3chb4
    ENDIF
  END
END

q3chb3w
  RANGE 1 490
  LEGAL
    998
    999
  END
  MUSTENTER
END

q3chb4
  RANGE 3 99
  MUSTENTER
```

8. INDITN.CHK

```
END

q3chb5
  RANGE 3 110
  LEGAL
    998
    999
  END
  MUSTENTER
  AFTER ENTRY
    IF (q3chb5<>998) AND (q3chb5>age) THEN
      HELP "q3chb5 cannot be more than age"
      CLEAR q3chb5
      GOTO q3chb5
    ENDIF
    IF (q3chb2=2)and (q3chb5<998) and (q3chb4<98) THEN
      q3chb6=q3chb5-q3chb4
    ENDIF
    IF (q3chb2=1)and (age<998) and (q3chb4<98) THEN
      q3chb6=age-q3chb4
    ENDIF
  END
END

q3chb6
  NOENTER
  RANGE 0 99
END

q3chc
  COMMENT LEGAL USE label_q3sma SHOW
  MUSTENTER
  AFTER ENTRY
    IF q3chc=2 THEN
      q3chc1="not applicable"
      q3chc2=8
      q3chc3d=98
      q3chc3w=998
      q3chc4=98
      q3chc5=998
      q3chc6=98
      GOTO q3chd
    ENDIF
  END
END

q3chc1
  MUSTENTER
END

q3chc2
  COMMENT LEGAL USE label_q3sma2 SHOW
  MUSTENTER
  TYPE COMMENT
END

q3chc3d
  RANGE 1 70
```

8. INDITN.CHK

```
  LEGAL
    98
    99
  END
  MUSTENTER
  AFTER ENTRY
    IF q3chc3d<71 THEN
      q3chc3w=998
      GOTO q3chc4
    ENDIF
  END
END

q3chc3w
  RANGE 1 490
  LEGAL
    998
    999
  END
  MUSTENTER
END

q3chc4
  RANGE 3 99
  MUSTENTER
END

q3chc5
  RANGE 3 110
  LEGAL
    998
    999
  END
  MUSTENTER
  AFTER ENTRY
    IF (q3chc5<>998) AND (q3chc5>age) THEN
      HELP "q3chc5 cannot be more than age"
      CLEAR q3chc5
      GOTO q3chc5
    ENDIF
    IF (q3chc2=2)and (q3chc5<998) and (q3chc4<98) THEN
      q3chc6=q3chc5-q3chc4
    ENDIF
    IF (q3chc2=1)and (age<998) and (q3chc4<98) THEN
      q3chc6=age-q3chc4
    ENDIF
  END
END

q3chc6
  NOENTER
  RANGE 0 99
END

q3chd
  COMMENT LEGAL USE label_q3sma SHOW
  MUSTENTER
  AFTER ENTRY
```

8. INDITN.CHK

```
    IF (q3cha=2) AND (q3chb=2) AND (q3chc=2) AND (q3chd=2) THEN
      q19=8
      q20="not applicable"
      q20a=9999.98
      q20b=9999.98
    ENDIF
    IF q3chd=2 THEN
      q3chd1="not applicable"
      q3chd2=8
      q3chd3d=98
      q3chd3w=998
      q3chd4=98
      q3chd5=998
      q3chd6=98
      GOTO q3sna
    ENDIF
  END
END

q3chd1
  MUSTENTER
END

q3chd2
  COMMENT LEGAL USE label_q3sma2 SHOW
  MUSTENTER
  TYPE COMMENT
END

q3chd3d
  RANGE 1 70
  LEGAL
    98
    99
  END
  MUSTENTER
  AFTER ENTRY
    IF q3chd3d<71 THEN
      q3chd3w=998
      GOTO q3chd4
    ENDIF
  END
END

q3chd3w
  RANGE 1 490
  LEGAL
    998
    999
  END
  MUSTENTER
END

q3chd4
  RANGE 3 99
  MUSTENTER
END
```

8. INDITN.CHK

```
q3chd5
  RANGE 3 110
  LEGAL
    998
    999
  END
  MUSTENTER
  AFTER ENTRY
    IF (q3chd5<>998) AND (q3chd5>age) THEN
      HELP "q3chd5 cannot be more than age"
      CLEAR q3chd5
      GOTO q3chd5
    ENDIF
    IF (q3chd2=2)and (q3chd5<998) and (q3chd4<98) THEN
      q3chd6=q3chd5-q3chd4
    ENDIF
    IF (q3chd2=1)and (age<998) and (q3chd4<98) THEN
      q3chd6=age-q3chd4
    ENDIF
  END
END

q3chd6
  NOENTER
  RANGE 0 99
END

q3sna
  COMMENT LEGAL USE label_q3sma SHOW
  MUSTENTER
  AFTER ENTRY
    IF q3sna=2 THEN
      q3sna1="not applicable"
      q3sna2=8
      q3sna3d=98
      q3sna3w=998
      q3sna4=98
      q3sna5=998
      q3sna6=98
      GOTO q3snb
    ENDIF
  END
END

q3sna1
  COMMENT LEGAL USE label_q3sna1 SHOW
  MUSTENTER
  TYPE COMMENT
END

q3sna2
  COMMENT LEGAL USE label_q3sma2 SHOW
  MUSTENTER
  TYPE COMMENT
END

q3sna3d
  RANGE 1 70
```

8. INDITN.CHK

```
  LEGAL
    98
    99
  END
  MUSTENTER
  AFTER ENTRY
    IF q3sna3d<71 THEN
      q3sna3w=998
      GOTO q3sna4
    ENDIF
  END
END

q3sna3w
  RANGE 1 490
  LEGAL
    998
    999
  END
  MUSTENTER
END

q3sna4
  RANGE 3 99
  MUSTENTER
END

q3sna5
  RANGE 3 110
  LEGAL
    998
    999
  END
  MUSTENTER
  AFTER ENTRY
    IF (q3sna5<>998) AND (q3sna5>age) THEN
      HELP "q3sna5 cannot be more than age"
      CLEAR q3sna5
      GOTO q3sna5
    ENDIF
    IF (q3sna2=2)and (q3sna5<998) and (q3sna4<98) THEN
      q3sna6=q3sna5-q3sna4
    ENDIF
    IF (q3sna2=1)and (age<998) and (q3sna4<98) THEN
      q3sna6=age-q3sna4
    ENDIF
  END
END

q3sna6
  NOENTER
  RANGE 0 99
END

q3snb
  COMMENT LEGAL USE label_q3sma SHOW
  MUSTENTER
  AFTER ENTRY
```

8. INDITN.CHK

```
    IF q3snb=2 THEN
      q3snb1="not applicable"
      q3snb2=8
      q3snb3d=98
      q3snb3w=998
      q3snb4=98
      q3snb5=998
      q3snb6=98
      GOTO q3a1a
    ENDIF
  END
END

q3snb1
  COMMENT LEGAL USE label_q3sna1 SHOW
  MUSTENTER
  TYPE COMMENT
END

q3snb2
  COMMENT LEGAL USE label_q3snb2 SHOW
  MUSTENTER
  TYPE COMMENT
END

q3snb3d
  RANGE 1 70
  LEGAL
    98
    99
  END
  MUSTENTER
  AFTER ENTRY
    IF q3snb3d<71 THEN
      q3snb3w=998
      GOTO q3snb4
    ENDIF
  END
END

q3snb3w
  RANGE 1 490
  LEGAL
    998
    999
  END
  MUSTENTER
END

q3snb4
  RANGE 3 99
  MUSTENTER
END

q3snb5
  RANGE 3 110
  LEGAL
    998
```

8. INDITN.CHK

```
    999
  END
  MUSTENTER
  AFTER ENTRY
    IF (q3snb5<>998) AND (q3snb5>age) THEN
      HELP "q3snb5 cannot be more than age"
      CLEAR q3snb5
      GOTO q3snb5
    ENDIF
    IF (q3snb2=2)and (q3snb5<998) and (q3snb4<98) THEN
      q3snb6=q3snb5-q3snb4
    ENDIF
    IF (q3snb2=1)and (age<998) and (q3snb4<98) THEN
      q3snb6=age-q3snb4
    ENDIF
  END
END

q3snb6
  NOENTER
  RANGE 0 99
END

q3ala
  COMMENT LEGAL USE label_q3sma SHOW
  MUSTENTER
  AFTER ENTRY
    IF q3ala=2 THEN
      q3ala1="not applicable"
      q3ala2=8
      q3ala3d=98
      q3ala3w=998
      q3ala4=98
      q3ala5=998
      q3ala6=98
      GOTO q4
    ENDIF
  END
END

q3ala1
  MUSTENTER
END

q3ala2
  COMMENT LEGAL USE label_q3snb2 SHOW
  MUSTENTER
  TYPE COMMENT
END

q3ala3d
  RANGE 1 70
  LEGAL
    98
    99
  END
  MUSTENTER
  AFTER ENTRY
```

8. INDITN.CHK

```
    IF q3ala3d<71 THEN
      q3ala3w=998
      GOTO q3ala4
    ENDIF
  END
END

q3ala3w
  RANGE 1 490
  LEGAL
    998
    999
  END
  MUSTENTER
END

q3ala4
  RANGE 3 99
  MUSTENTER
END

q3ala5
  RANGE 3 110
  LEGAL
    998
    999
  END
  MUSTENTER
  AFTER ENTRY
    IF (q3ala5<>998) AND (q3ala5>age) THEN
      HELP "q3ala5 cannot be more than age"
      CLEAR q3ala5
      GOTO q3ala5
    ENDIF
    IF (q3ala2=2)and (q3ala5<998) and (q3ala4<98) THEN
      q3ala6=q3ala5-q3ala4
    ENDIF
    IF (q3ala2=1)and (age<998) and (q3ala4<98) THEN
      q3ala6=age-q3ala4
    ENDIF
  END
END

q3ala6
  NOENTER
  RANGE 0 99
END

q4
  COMMENT LEGAL USE label_q4 SHOW
  MUSTENTER
  TYPE COMMENT
  AFTER ENTRY
    HELP "if Q2= 1 (past user-yes), then q4 to q20 should be entered as
not applicable and then continue entering from Q21"
  END
END
```

8. INDITN.CHK

```
q5
  RANGE 0 99
  MUSTENTER
END

q5a
  RANGE 1 50
  LEGAL
    98
    99
  END
  MUSTENTER
END

q6
  RANGE 0.01 9999.99
  MUSTENTER
END

q7
  RANGE 1 999999999.99
  MUSTENTER
END

q8
  COMMENT LEGAL USE label_q2 SHOW
  MUSTENTER
  TYPE COMMENT
END

q9
  COMMENT LEGAL USE label_q1 SHOW
  MUSTENTER
  TYPE COMMENT
END

q10
  COMMENT LEGAL USE label_q1 SHOW
  MUSTENTER
  TYPE COMMENT
END

q11
  COMMENT LEGAL USE label_q1 SHOW
  MUSTENTER
  TYPE COMMENT
END

q12
  COMMENT LEGAL USE label_q1 SHOW
  MUSTENTER
  TYPE COMMENT
END

q13
  COMMENT LEGAL USE label_q1 SHOW
  MUSTENTER
  TYPE COMMENT
```

```
END

q148
  COMMENT LEGAL USE label_q1 SHOW
  MUSTENTER
  TYPE COMMENT
  AFTER ENTRY
    IF q148=1 THEN
      q141=8
      q142=8
      q143=8
      q144=8
      q145=8
      q146=8
      q147=8
      Q149=8
      q15=8
      GOTO q16
    ENDIF
  END
END

q141
  COMMENT LEGAL USE label_q1 SHOW
  MUSTENTER
  TYPE COMMENT
END

q142
  COMMENT LEGAL USE label_q1 SHOW
  MUSTENTER
  TYPE COMMENT
END

q143
  COMMENT LEGAL USE label_q1 SHOW
  MUSTENTER
  TYPE COMMENT
END

q144
  COMMENT LEGAL USE label_q1 SHOW
  MUSTENTER
  TYPE COMMENT
END

q145
  COMMENT LEGAL USE label_q1 SHOW
  MUSTENTER
  TYPE COMMENT
END

q146
  COMMENT LEGAL USE label_q1 SHOW
  MUSTENTER
  TYPE COMMENT
END
```

8. INDITN.CHK

```
q147
  COMMENT LEGAL USE label_q1 SHOW
  MUSTENTER
  TYPE COMMENT
END

q149
  COMMENT LEGAL USE label_q1 SHOW
  MUSTENTER
  TYPE COMMENT
END

q15
  COMMENT LEGAL USE label_q15 SHOW
  MUSTENTER
  TYPE COMMENT
END

q16
  COMMENT LEGAL USE label_q16 SHOW
  MUSTENTER
  TYPE COMMENT
END

q17
  COMMENT LEGAL USE label_q17 SHOW
  MUSTENTER
  TYPE COMMENT
END

q18
  COMMENT LEGAL USE label_q1 SHOW
  MUSTENTER
  TYPE COMMENT
END

q19
  COMMENT LEGAL USE label_q1 SHOW
  MUSTENTER
  TYPE COMMENT
END

q20
  MUSTENTER
END

q20a
  MUSTENTER
END

q20b
  MUSTENTER
END

q21
  COMMENT LEGAL USE label_q1 SHOW
  MUSTENTER
  TYPE COMMENT
```

```
END

q22
  COMMENT LEGAL USE label_q22 SHOW
  MUSTENTER
  TYPE COMMENT
END

q23a
  COMMENT LEGAL USE label_q1 SHOW
  MUSTENTER
  TYPE COMMENT
END

q23b
  COMMENT LEGAL USE label_q23b SHOW
  MUSTENTER
  TYPE COMMENT
END

q24
  COMMENT LEGAL USE label_q24 SHOW
  MUSTENTER
  TYPE COMMENT
END

q25
  COMMENT LEGAL USE label_q25 SHOW
  MUSTENTER
  TYPE COMMENT
END

q26
  COMMENT LEGAL USE label_q25 SHOW
  MUSTENTER
  TYPE COMMENT
END

q271
  COMMENT LEGAL USE label_q1 SHOW
  MUSTENTER
  TYPE COMMENT
END

q272
  COMMENT LEGAL USE label_q1 SHOW
  MUSTENTER
  TYPE COMMENT
END

q273
  COMMENT LEGAL USE label_q1 SHOW
  MUSTENTER
  TYPE COMMENT
END

q274
  COMMENT LEGAL USE label_q1 SHOW
```

```
  MUSTENTER
  TYPE COMMENT
END

q275
  COMMENT LEGAL USE label_q1 SHOW
  MUSTENTER
  TYPE COMMENT
END

q276
  COMMENT LEGAL USE label_q1 SHOW
  MUSTENTER
  TYPE COMMENT
END

q288
  COMMENT LEGAL USE label_q1 SHOW
  MUSTENTER
  TYPE COMMENT
  AFTER ENTRY
    IF q288=1 THEN
      q281=8
      q282=8
      q283=8
      q284=8
      q285=8
      q286=8
      q287=8
      GOTO q298
    ENDIF
  END
END

q281
  COMMENT LEGAL USE label_q1 SHOW
  MUSTENTER
  TYPE COMMENT
END

q282
  COMMENT LEGAL USE label_q1 SHOW
  MUSTENTER
  TYPE COMMENT
END

q283
  COMMENT LEGAL USE label_q1 SHOW
  MUSTENTER
  TYPE COMMENT
END

q284
  COMMENT LEGAL USE label_q1 SHOW
  MUSTENTER
  TYPE COMMENT
END
```

8. INDITN.CHK

```
q285
  COMMENT LEGAL USE label_q1 SHOW
  MUSTENTER
  TYPE COMMENT
END

q286
  COMMENT LEGAL USE label_q1 SHOW
  MUSTENTER
  TYPE COMMENT
END

q287
  COMMENT LEGAL USE label_q1 SHOW
  MUSTENTER
  TYPE COMMENT
END

q298
  COMMENT LEGAL USE label_q1 SHOW
  MUSTENTER
  TYPE COMMENT
  AFTER ENTRY
    IF q298=1 THEN
      q291=8
      q292=8
      q293=8
      q294=8
      q295=8
      q296=8
      q297=8
      GOTO q30
    ENDIF
  END
END

q291
  COMMENT LEGAL USE label_q1 SHOW
  MUSTENTER
  TYPE COMMENT
END

q292
  COMMENT LEGAL USE label_q1 SHOW
  MUSTENTER
  TYPE COMMENT
END

q293
  COMMENT LEGAL USE label_q1 SHOW
  MUSTENTER
  TYPE COMMENT
END

q294
  COMMENT LEGAL USE label_q1 SHOW
  MUSTENTER
  TYPE COMMENT
```

8. INDITN.CHK

```
END

q295
  COMMENT LEGAL USE label_q1 SHOW
  MUSTENTER
  TYPE COMMENT
END

q296
  COMMENT LEGAL USE label_q1 SHOW
  MUSTENTER
  TYPE COMMENT
END

q297
  COMMENT LEGAL USE label_q1 SHOW
  MUSTENTER
  TYPE COMMENT
END

q30
  COMMENT LEGAL USE label_q30 SHOW
  MUSTENTER
  TYPE COMMENT
END
```

9. INDITN.REC

```
289 1 VLAB ~kq:CM89s2jGP9H=:kq~ Filelabel: TNTS-INDIVIDUAL
_label1        1   1  30   0   0   0   0 112 Study name: Tamil Nadu
Tobacco Survey
_label2        1   2  30   0   0   0   0 112 Dear Data entry operator, Do
not use mouse or key pad during data entry.
_label3        1   3  30   0   0   0   0 112 Use enter/tab/arrow key(s)
to move from one field to another
_label4        1   4  30   0   0   0   0 112 Press F9 if options do not
appear for categorical variable
_label5        1   5  30   0   0   0   0 112 Keep a printout of data
documentation sheet by your side during data entry
_label6        1   6  30   0   0   0   0 112 Contact Mr Prabhakar in case
of any query during data entry 99622 76628
#secs          1   8  30  81   8   0   4 112 secs
Time in seconds to fill this record
_label7       85   8  30   0   0   0   0 112  noenter
_idindi        1   9  30  81   9   1  18 112 idindi
unique id for individual
_label8       99   9  30   0   0   0   0 112  unique id, no enter field
_idhouse       1  10  30  81  10   1  11 112 idhouse
Unique id for the household
_label9       92  10  30   0   0   0   0 112  key id, noenter field
_distcode      1  11  30  81  11   1   6 112 distcode
district code
_label10      87  11  30   0   0   0   0 112  noenter field
_distcopy      1  12  30  81  12   1  40 112 distcopy
Copy of the distarea
_label11     121  12  30   0   0   0   0 112  noenter field
_pass          1  13  30  81  13  18  12 119 pass
code
_deo           1  15  30  81  15   1  20 112 deo
name of dataentry operator
_distarea      1  16  30  81  16   1  40 112 distarea
name of the district including area
#hhno          1  17  30  81  17   0   4 112 hhno
household serial number
_label12      85  17  30   0   0   0   0 112  enter 2001, 2002, 2003
onwards if missing/non unique, mention the same in quest
_label13     165  17  30   0   0   0   0 112 ionnaire, press F5 to make a
note
_indino        1  18  30  81  18   1  10 112 indino
individual participant id no
_label14      91  18  30   0   0   0   0 112  use appropriate id in case
of missing esp those >15y olds who do not give inter
_label15     171  18  30   0   0   0   0 112 view
_label16       1  20  30   0   0   0   0 112 Individual Questionnaire
_name          1  21  30  81  21   1  40 112 name
Participant's name
#gender        1  22  30  81  22   0   1 112 gender
participant's gender
#age           1  23  30  81  23   0   3 112 age
participant's age
_label17      84  23  30   0   0   0   0 112  enter 999 if the data is
not recorded
#occ           1  24  30  81  24   0   1 112 occ
participant's occupation
#edn           1  25  30  81  25   0   1 112 edn
participant's education
```

9. INDITN.REC

```
#phno          1  26  30  81  26   6  10 112 phno
phone number
_label18      91  26  30   0   0   0   0 112  enter 99999999999 (10
digit) for missing, do not enter 0 or +91 before the numb
_label19     171  26  30   0   0   0   0 112 er
#filled        1  28  30  81  28   0   1 112 filled
was questionnaire filled
#reason        1  29  30  81  29   0   1 112 reason
why was the questionnaire not filled
#q1            1  31  30  81  31   0   1 112 Q1
Do you currently use tobacco?
#q2            1  32  30  81  32   0   1 112 Q2
In the past, have you used tobacco?
_label20       1  34  30   0   0   0   0 112 Smoking and Chewing Habits
#q3sma         1  35  30  81  35   0   1 112 Q3sma        whether user of
tobacco smoking Cigarettes (filtered/non-filtered)
_label21      82  35  30   0   0   0   0 112  mention yes if row in Q3
table is filled. This variable is only for efficient d
_label22     162  35  30   0   0   0   0 112 ata entry, not to be
used/included in analysis
_q3sma1        1  36  30  81  36   1  18 112 Q3sma1           name of the
tobacco smoking Cigarettes (filtered/non-filtered)
_label23      99  36  30   0   0   0   0 112  mention not applicable if
required
#q3sma2        1  37  30  81  37   0   1 112 Q3sma2
cigarettes (filtered/non-filtered) smoking Current/Past
#q3sma3d       1  38  30  81  38   0   2 112 Q3sma3d
cigarettes (filtered/non-filtered)daily frequency
_label24      83  38  30   0   0   0   0 112  enter 99 if the data is not
recorded, 98 if not applicable
#q3sma3w       1  39  30  81  39   0   3 112 Q3sma3w
cigarettes (filtered/non-filtered)weekly frequency
_label25      84  39  30   0   0   0   0 112  enter 999 if the data is
not recorded, 998 if not applicable
#q3sma4        1  40  30  81  40   0   2 112 Q3sma4
cigarettes (filtered/non-filtered) age started in
_label26      83  40  30   0   0   0   0 112  enter 99 if the data is not
recorded, 98 if not applicable
#q3sma5        1  41  30  81  41   0   3 112 Q3sma5
cigarettes (filtered/non-filtered) age past user stopped at
_label27      84  41  30   0   0   0   0 112  enter 999 if the data is
not recorded, 998 if not applicable
#q3sma6        1  42  30  81  42   0   2 112 Q3sma6
cigarettes (filtered/non-filtered) duration of use
_label28      83  42  30   0   0   0   0 112  noenter
#q3smb         1  44  30  81  44   0   1 112 Q3smb
whether user of tobacco smoking bidi
_label29      82  44  30   0   0   0   0 112  mention yes if row in Q3
table is filled. This variable is only for efficient d
_label30     162  44  30   0   0   0   0 112 ata entry, not to be
used/included in analysis
_q3smb1        1  45  30  81  45   1  18 112 Q3smb1
name of the tobacco smoking bidi brand
_label31      99  45  30   0   0   0   0 112  mention not applicable if
required
#q3smb2        1  46  30  81  46   0   1 112 Q3smb2
bidi smoking Current/Past
```

9. INDITN.REC

```
#q3smb3d       1  47  30  81  47   0   2 112 Q3smb3d
bidi smoking daily frequency
_label32      83  47  30   0   0   0   0 112  enter 99 if the data is not
recorded, 98 if not applicable
#q3smb3w       1  48  30  81  48   0   3 112 Q3smb3w
bidi smoking weekly frequency
_label33      84  48  30   0   0   0   0 112  enter 999 if the data is
not recorded, 998 if not applicable
#q3smb4        1  49  30  81  49   0   2 112 Q3smb4
bidi smoking age started in
_label34      83  49  30   0   0   0   0 112  enter 99 if the data is not
recorded, 98 if not applicable
#q3smb5        1  50  30  81  50   0   3 112 Q3smb5
bidi smoking age past user stopped at
_label35      84  50  30   0   0   0   0 112  enter 999 if the data is
not recorded, 998 if not applicable
#q3smb6        1  51  30  81  51   0   2 112 Q3smb6
bidi smoking duration of use
_label36      83  51  30   0   0   0   0 112  noenter
#q3smc         1  53  30  81  53   0   1 112 Q3smc
whether user of tobacco smoking Chutta/Cigar/Cheroot
_label37      82  53  30   0   0   0   0 112  mention yes if row in Q3
table is filled. This variable is only for efficient d
_label38     162  53  30   0   0   0   0 112 ata entry, not to be
used/included in analysis
_q3smc1        1  54  30  81  54   1  18 112 Q3smc1
name of the tobacco smoking Chutta/Cigar/Cheroot brand
_label39      99  54  30   0   0   0   0 112  mention not applicable if
required
#q3smc2        1  55  30  81  55   0   1 112 Q3smc2
Chutta/Cigar/Cheroot smoking Current/Past
#q3smc3d       1  56  30  81  56   0   2 112 Q3smc3d
Chutta/Cigar/Cheroot smoking daily frequency
_label40      83  56  30   0   0   0   0 112  enter 99 if the data is not
recorded, 98 if not applicable
#q3smc3w       1  57  30  81  57   0   3 112 Q3smc3w
Chutta/Cigar/Cheroot smoking weekly frequency
_label41      84  57  30   0   0   0   0 112  enter 999 if the data is
not recorded, 998 if not applicable
#q3smc4        1  58  30  81  58   0   2 112 Q3smc4
Chutta/Cigar/Cheroot smoking age started in
_label42      83  58  30   0   0   0   0 112  enter 99 if the data is not
recorded, 98 if not applicable
#q3smc5        1  59  30  81  59   0   3 112 Q3smc5
Chutta/Cigar/Cheroot smoking age past user stopped at
_label43      84  59  30   0   0   0   0 112  enter 999 if the data is
not recorded, 998 if not applicable
#q3smc6        1  60  30  81  60   0   2 112 Q3smc6
Chutta/Cigar/Cheroot smoking duration of use
_label44      83  60  30   0   0   0   0 112  noenter
#q3smd         1  62  30  81  62   0   1 112 Q3smd
whether user of tobacco smoking others
_label45      82  62  30   0   0   0   0 112  mention yes if row in Q3
table is filled. This variable is only for efficient d
_label46     162  62  30   0   0   0   0 112 ata entry, not to be
used/included in analysis
_q3smd1        1  63  30  81  63   1  18 112 Q3smd1
name of the tobacco smoking Others brand
```

```
_label47      99  63  30   0   0   0   0 112  mention not applicable if
required
#q3smd2        1  64  30  81  64   0   1 112 Q3smd2
Others smoking Current/Past
#q3smd3d       1  65  30  81  65   0   2 112 Q3smd3d
Others smoking daily frequency
_label48      83  65  30   0   0   0   0 112  enter 99 if the data is not
recorded, 98 if not applicable
#q3smd3w       1  66  30  81  66   0   3 112 Q3smd3w
Others smoking weekly frequency
_label49      84  66  30   0   0   0   0 112  enter 999 if the data is
not recorded, 998 if not applicable
#q3smd4        1  67  30  81  67   0   2 112 Q3smd4
Others smoking age started in
_label50      83  67  30   0   0   0   0 112  enter 99 if the data is not
recorded, 98 if not applicable
#q3smd5        1  68  30  81  68   0   3 112 Q3smd5
Others smoking age past user stopped at
_label51      84  68  30   0   0   0   0 112  enter 999 if the data is
not recorded, 998 if not applicable
#q3smd6        1  69  30  81  69   0   2 112 Q3smd6
Others smoking duration of use
_label52      83  69  30   0   0   0   0 112  noenter
#q3cha         1  71  30  81  71   0   1 112 Q3cha
whether user of tobacco Chewing alone
_label53      82  71  30   0   0   0   0 112  mention yes if row in Q3
table is filled. This variable is only for efficient d
_label54     162  71  30   0   0   0   0 112 ata entry, not to be
used/included in analysis
_q3cha1        1  72  30  81  72   1  18 112 Q3cha1
name of the chewing tobacco alone
_label55      99  72  30   0   0   0   0 112  mention not applicable if
required
#q3cha2        1  73  30  81  73   0   1 112 Q3cha2
tobacco alone chewing Current/Past
#q3cha3d       1  74  30  81  74   0   2 112 Q3cha3d
tobacco alone chewing daily frequency
_label56      83  74  30   0   0   0   0 112  enter 99 if the data is not
recorded, 98 if not applicable
#q3cha3w       1  75  30  81  75   0   3 112 Q3cha3w
tobacco alone chewing weekly frequency
_label57      84  75  30   0   0   0   0 112  enter 999 if the data is
not recorded, 998 if not applicable
#q3cha4        1  76  30  81  76   0   2 112 Q3cha4
tobacco alone chewing age started in
_label58      83  76  30   0   0   0   0 112  enter 99 if the data is not
recorded, 98 if not applicable
#q3cha5        1  77  30  81  77   0   3 112 Q3cha5
tobacco alone chewing past user stopped at
_label59      84  77  30   0   0   0   0 112  enter 999 if the data is
not recorded, 998 if not applicable
#q3cha6        1  78  30  81  78   0   2 112 Q3cha6
tobacco alone chewing duration of use
_label60      83  78  30   0   0   0   0 112  noenter
#q3chb         1  80  30  81  80   0   1 112 Q3chb
whether user of chewing TOBACCO/AN/PAN
_label61      82  80  30   0   0   0   0 112  mention yes if row in Q3
table is filled. This variable is only for efficient d
```

```
_label62     162  80  30   0   0   0   0 112 ata entry, not to be
used/included in analysis
_q3chb1        1  81  30  81  81   1  18 112 Q3chb1
name of the chewing TOBACCO/AN/PAN
_label63      99  81  30   0   0   0   0 112  mention not applicable if
required
#q3chb2        1  82  30  81  82   0   1 112 Q3chb2
TOBACCO/AN/PAN chewing Current/Past
#q3chb3d       1  83  30  81  83   0   2 112 Q3chb3d
TOBACCO/AN/PAN chewing daily frequency
_label64      83  83  30   0   0   0   0 112  enter 99 if the data is not
recorded, 98 if not applicable
#q3chb3w       1  84  30  81  84   0   3 112 Q3chb3w
TOBACCO/AN/PAN chewing weekly frequency
_label65      84  84  30   0   0   0   0 112  enter 999 if the data is
not recorded, 998 if not applicable
#q3chb4        1  85  30  81  85   0   2 112 Q3chb4
TOBACCO/AN/PAN chewing age started in
_label66      83  85  30   0   0   0   0 112  enter 99 if the data is not
recorded, 98 if not applicable
#q3chb5        1  86  30  81  86   0   3 112 Q3chb5
TOBACCO/AN/PAN chewing past user stopped at
_label67      84  86  30   0   0   0   0 112  enter 999 if the data is
not recorded, 998 if not applicable
#q3chb6        1  87  30  81  87   0   2 112 Q3chb6
TOBACCO/AN/PAN chewing duration of use
_label68      83  87  30   0   0   0   0 112  noenter
#q3chc         1  89  30  81  89   0   1 112 Q3chc
whether user of chewing Tobacco+Pan
_label69      82  89  30   0   0   0   0 112  mention yes if row in Q3
table is filled. This variable is only for efficient d
_label70     162  89  30   0   0   0   0 112 ata entry, not to be
used/included in analysis
_q3chc1        1  90  30  81  90   1  18 112 Q3chc1
name of the chewing Tobacco+Pan
_label71      99  90  30   0   0   0   0 112  mention not applicable if
required
#q3chc2        1  91  30  81  91   0   1 112 Q3chc2
Tobacco+Pan  chewing Current/Past
#q3chc3d       1  92  30  81  92   0   2 112 Q3chc3d
Tobacco+Pan  chewing daily frequency
_label72      83  92  30   0   0   0   0 112  enter 99 if the data is not
recorded, 98 if not applicable
#q3chc3w       1  93  30  81  93   0   3 112 Q3chc3w
Tobacco+Pan chewing weekly frequency
_label73      84  93  30   0   0   0   0 112  enter 999 if the data is
not recorded, 998 if not applicable
#q3chc4        1  94  30  81  94   0   2 112 Q3chc4
Tobacco+Pan  chewing age started in
_label74      83  94  30   0   0   0   0 112  enter 99 if the data is not
recorded, 98 if not applicable
#q3chc5        1  95  30  81  95   0   3 112 Q3chc5
Tobacco+Pan  chewing past user stopped at
_label75      84  95  30   0   0   0   0 112  enter 999 if the data is
not recorded, 998 if not applicable
#q3chc6        1  96  30  81  96   0   2 112 Q3chc6
Tobacco+Pan duration of use
_label76      83  96  30   0   0   0   0 112  noenter
```

```
#q3chd         1  98  30  81  98   0   1 112 Q3chd
whether user of chewing tobacco,others
_label77      82  98  30   0   0   0   0 112  mention yes if row in Q3
table is filled. This variable is only for efficient d
_label78     162  98  30   0   0   0   0 112 ata entry, not to be
used/included in analysis
_q3chd1        1  99  30  81  99   1  18 112 Q3chd1
name of the chewing tobacco,others
_label79      99  99  30   0   0   0   0 112  mention not applicable if
required
#q3chd2        1 100  30  81 100   0   1 112 Q3chd2
tobacco,others chewing Current/Past
#q3chd3d       1 101  30  81 101   0   2 112 Q3chd3d
tobacco,others chewing daily frequency
_label80      83 101  30   0   0   0   0 112  enter 99 if the data is not
recorded, 98 if not applicable
#q3chd3w       1 102  30  81 102   0   3 112 Q3chd3w
tobacco,others chewing weekly frequency
_label81      84 102  30   0   0   0   0 112  enter 999 if the data is
not recorded, 998 if not applicable
#q3chd4        1 103  30  81 103   0   2 112 Q3chd4
tobacco,others chewing age started in
_label82      83 103  30   0   0   0   0 112  enter 99 if the data is not
recorded, 98 if not applicable
#q3chd5        1 104  30  81 104   0   3 112 Q3chd5
tobacco,others chewing past user stopped at
_label83      84 104  30   0   0   0   0 112  enter 999 if the data is
not recorded, 998 if not applicable
#q3chd6        1 105  30  81 105   0   2 112 Q3chd6
tobacco,others chewing duration of use
_label84      83 105  30   0   0   0   0 112  noenter
#q3sna         1 107  30  81 107   0   1 112 Q3sna
whether user of snuffing tobacco, by mouth
_label85      82 107  30   0   0   0   0 112  mention yes if row in Q3
table is filled. This variable is only for efficient d
_label86     162 107  30   0   0   0   0 112 ata entry, not to be
used/included in analysis
_q3sna1        1 108  30  81 108   1  18 112 Q3sna1
name of the snuffing tobacco, by mouth
_label87      99 108  30   0   0   0   0 112  mention not applicable if
required
#q3sna2        1 109  30  81 109   0   1 112 Q3sna2
snuffing by mouth Current/Past
#q3sna3d       1 110  30  81 110   0   2 112 Q3sna3d
snuffing by mouth daily frequency
_label88      83 110  30   0   0   0   0 112  enter 99 if the data is not
recorded, 98 if not applicable
#q3sna3w       1 111  30  81 111   0   3 112 Q3sna3w
snuffing by mouth weekly frequency
_label89      84 111  30   0   0   0   0 112  enter 999 if the data is
not recorded, 998 if not applicable
#q3sna4        1 112  30  81 112   0   2 112 Q3sna4
snuffing by mouth age started in
_label90      83 112  30   0   0   0   0 112  enter 99 if the data is not
recorded, 98 if not applicable
#q3sna5        1 113  30  81 113   0   3 112 Q3sna5
snuffing by mouth past user stopped at
```

9. INDITN.REC

```
_label91      84 113  30   0   0   0   0 112  enter 999 if the data is
not recorded, 998 if not applicable
#q3sna6        1 114  30  81 114   0   2 112 Q3sna6
snuffing by mouth duration of use
_label92      83 114  30   0   0   0   0 112  noenter
#q3snb         1 116  30  81 116   0   1 112 Q3snb
whether user of snuffing tobacco, by nose
_label93      82 116  30   0   0   0   0 112  mention yes if row in Q3
table is filled. This variable is only for efficient d
_label94     162 116  30   0   0   0   0 112 ata entry, not to be
used/included in analysis
_q3snb1        1 117  30  81 117   1  18 112 Q3snb1
name of the snuffing tobacco, by nose
_label95      99 117  30   0   0   0   0 112  mention not applicable if
required
#q3snb2        1 118  30  81 118   0   1 112 Q3snb2
snuffing by nose Current/Past
#q3snb3d       1 119  30  81 119   0   2 112 Q3snb3d
snuffing by nose daily frequency
_label96      83 119  30   0   0   0   0 112  enter 99 if the data is not
recorded, 98 if not applicable
#q3snb3w       1 120  30  81 120   0   3 112 Q3snb3w
snuffing by nose weekly frequency
_label97      84 120  30   0   0   0   0 112  enter 999 if the data is
not recorded, 998 if not applicable
#q3snb4        1 121  30  81 121   0   2 112 Q3snb4
snuffing by nose age started in
_label98      83 121  30   0   0   0   0 112  enter 99 if the data is not
recorded, 98 if not applicable
#q3snb5        1 122  30  81 122   0   3 112 Q3snb5
snuffing by nose past user stopped at
_label99      84 122  30   0   0   0   0 112  enter 999 if the data is
not recorded, 998 if not applicable
#q3snb6        1 123  30  81 123   0   2 112 Q3snb6
snuffing by nose duration of use
_label100     83 123  30   0   0   0   0 112  noenter
#q3ala         1 125  30  81 125   0   1 112 Q3ala
whether user of alcohol
_label101     82 125  30   0   0   0   0 112  mention yes if row in Q3
table is filled. This variable is only for efficient d
_label102    162 125  30   0   0   0   0 112 ata entry, not to be
used/included in analysis
_q3ala1        1 126  30  81 126   1  18 112 Q3ala1
name of the alcohol use
_label103     99 126  30   0   0   0   0 112  mention not applicable if
required
#q3ala2        1 127  30  81 127   0   1 112 Q3ala2
alcohol Current/Past
#q3ala3d       1 128  30  81 128   0   2 112 Q3ala3d
alcohol daily frequency
_label104     83 128  30   0   0   0   0 112  enter 99 if the data is not
recorded, 98 if not applicable
#q3ala3w       1 129  30  81 129   0   3 112 Q3ala3w
alcohol weekly frequency
_label105     84 129  30   0   0   0   0 112  enter 999 if the data is
not recorded, 998 if not applicable
#q3ala4        1 130  30  81 130   0   2 112 Q3ala4
alcohol age started in
```

9. INDITN.REC

```
_label106     83 130  30   0   0   0   0 112  enter 99 if the data is not
recorded, 98 if not applicable
#q3ala5        1 131  30  81 131   0   3 112 Q3ala5
alcohol past user stopped at
_label107     84 131  30   0   0   0   0 112  enter 999 if the data is
not recorded, 998 if not applicable
#q3ala6        1 132  30  81 132   0   2 112 Q3ala6
alcohol duration of use
_label108     83 132  30   0   0   0   0 112  noenter
#q4            1 134  30  81 134   0   1 112 Q4          post wake up,
when do you usually have your first smoke/chew/snuff?
#q5            1 135  30  81 135 101   4 112 Q5
number of cigarettes/Bidis/Packets purchased last time
_label109     85 135  30   0   0   0   0 112  99.9 not recorded, 99.8 not
applicable
#q5a           1 136  30  81 136   0   2 112 Q5a                  If it
is a pack, how many sticks were there in the packet?
_label110     83 136  30   0   0   0   0 112  99 not recorded, 98 not
applicable
#q6            1 137  30  81 137 102   7 112 Q6             In total, how
much money did you pay for this purchase (in INR)?
_label111     88 137  30   0   0   0   0 112  9999.99 not recorded,
9999.98 not applicable
#q7            1 138  30  81 138 102  12 112 Q7
total expense on this purchase (in INR) till date
_label112     93 138  30   0   0   0   0 112  9999.99 not recorded,
9999.98 not applicable
#q8            1 139  30  81 139   0   1 112 Q8
Are you concerned about your tobacco use?
#q9            1 140  30  81 140   0   1 112 Q9
Have you visited a doctor in the past 12 months?
#q10           1 141  30  81 141   0   1 112 Q10                     Did
the doctor ask if you use Tobacco (in last 12mths)?
#q11           1 142  30  81 142   0   1 112 Q11                      Did
the doctor advise to quit Tobacco(in last 12mths)?
#q12           1 143  30  81 143   0   1 112 Q12          Inlast
30days,didyou notice any warning on tobacco product youuse?
#q13           1 144  30  81 144   0   1 112 Q13          In last
30days,did warning labels led you to think about quitting?
_label113      1 145  30   0   0   0   0 112 Q14* In past 12 months,
which method did you try to stop using tobacco?
#q148          1 146  30  81 146   0   1 112 Q148
no attempt
#q141          1 147  30  81 147   0   1 112 Q141
counselling
#q142          1 148  30  81 148   0   1 112 Q142
nicotine replacement therapy
#q143          1 149  30  81 149   0   1 112 Q143
medications
#q144          1 150  30  81 150   0   1 112 Q144
switching to alternate tobacco
#q145          1 151  30  81 151   0   1 112 Q145
a quit line
#q146          1 152  30  81 152   0   1 112 Q146
traditional medicines
#q147          1 153  30  81 153   0   1 112 Q147
quit on my own
```

```
#q149           1 154  30  81 154   0   1 112 Q149
other method to quit
#q15            1 155  30  81 155   0   1 112 Q15           Ifyou have
quit completely,specify the method you found effective
#q16            1 156  30  81 156   0   1 112 Q16
How long did you stop using tobacco, since last quit?
#q17            1 157  30  81 157   0   1 112 Q17               Which of
the following best describes about quitting tobacco?
_label114       1 158  30   0   0   0   0 112 If less than 18 years
#q18            1 159  30  81 159   0   1 112 Q18              Were you
refused while purchasing tobacco in the past 30 days?
_label115       1 160  30   0   0   0   0 112 If Chewers
#q19            1 161  30  81 161   0   1 112 Q19               Did you
find it difficult to purchase tobacco in past 30days?
_q20            1 162  30  81 162   1  20 112 Q20
Brand name of the tobacco that is usually purchased
_label116     101 162  30   0   0   0   0 112  mention not applicable if
required
#q20a           1 163  30  81 163 102   7 112 Q20a              Price of
the tobacco brand (usually purchased)before the ban
_label117      88 163  30   0   0   0   0 112  9999.99 not recorded,
9999.98 not applicable
#q20b           1 164  30  81 164 102   7 112 Q20b               Price of
the tobacco brand (usually purchased)after the ban
_label118      88 164  30   0   0   0   0 112  9999.99 not recorded,
9999.98 not applicable
_label119       1 166  30   0   0   0   0 112 For Non-users as well as
users of tobacco in the household
#q21            1 168  30  81 168   0   1 112 Q21
Do you know the harmful effects of passive smoking?
#q22            1 169  30  81 169   0   1 112 Q22                 Which
describes the best rule for smoking inside your home?
#q23a           1 170  30  81 170   0   1 112 Q23a
Does anyone smoke inside home
#q23b           1 171  30  81 171   0   1 112 Q23b
How often does anyone smoke inside your home
#q24            1 172  30  81 172   0   1 112 Q24              Which best
describes the indoor smoking policy where you work?
#q25            1 173  30  81 173   0   1 112 Q25         During past
30days,did anyone smoke in indoor areas where you work?
#q26            1 174  30  81 174   0   1 112 Q26           Does
breathing,others smoke cause serious illness in Non-smokers?
_label120       1 175  30   0   0   0   0 112 Q27*   Does tobacco cause
the following (Based on what you know or believe)
#q271           1 176  30  81 176   0   1 112 Q271
stroke
#q272           1 177  30  81 177   0   1 112 Q272
heart attack
#q273           1 178  30  81 178   0   1 112 Q273
cancer
#q274           1 179  30  81 179   0   1 112 Q274
cause serious illness
#q275           1 180  30  81 180   0   1 112 Q275
infertility/impotence
#q276           1 181  30  81 181   0   1 112 Q276
don't know
_label121       1 182  30   0   0   0   0 112 Q28*    In last 30 days,any
information seen on tobacco danger or quitting?
```

```
#q288          1 183  30  81 183   0   1 112 Q288
not seen
#q281          1 184  30  81 184   0   1 112 Q281
television
#q282          1 185  30  81 185   0   1 112 Q282
radio
#q283          1 186  30  81 186   0   1 112 Q283
newspapers
#q284          1 187  30  81 187   0   1 112 Q284
billiboards
#q285          1 188  30  81 188   0   1 112 Q285
tobacco packs
#q286          1 189  30  81 189   0   1 112 Q286
movies
#q287          1 190  30  81 190   0   1 112 Q287
theatres
_label122      1 191  30   0   0   0   0 112 Q29* In last 30days,any
advertisement was seen promoting tobacco products?
#q298          1 192  30  81 192   0   1 112 Q298
not seen
#q291          1 193  30  81 193   0   1 112 Q291
television
#q292          1 194  30  81 194   0   1 112 Q292
radio
#q293          1 195  30  81 195   0   1 112 Q293
newspapers
#q294          1 196  30  81 196   0   1 112 Q294
billiboards
#q295          1 197  30  81 197   0   1 112 Q295
tobacco packs
#q296          1 198  30  81 198   0   1 112 Q296
movies
#q297          1 199  30  81 199   0   1 112 Q297
theatres
_q30           1 200  30  81 200   1  50 112 Q30                Do you
suggest any measures for curbing the use of tobacco?
_label123    131 200  30   0   0   0   0 112  mention "missing" if no
suggestion
```

10. SITE.CHK

```
v1
  KEY 1
END

v2
  KEY 2
END
```

11. SITE.REC

```
2 1 VLAB
_v1           1   1  30   5   1   1  20 112 v1
_v2           1   2  30   5   2   1   5 112 v2
Adyar chen non slum AYR_N!
Adyar chen slum     AYR_S!
Alandur chen non sluADR_N!
Alandur chen slum   ADR_S!
Ambattur chen non slABR_N!
Ambattur chen slum  ABR_S!
Annanagar chen non sANG_N!
Annanagar chen slum ANG_S!
Ariyalur rural      AR_R !
Ariyalur urban      AR_U !
Coimbattore rural   CO_R !
Coimbattore urban   CO_U !
Cuddalore rural     CU_R !
Cuddalore urban     CU_U !
Dharmapuri rural    DH_R !
Dharmapuri urban    DH_U !
Dindigul rural      DI_R !
Dindigul urban      DI_U !
Erode rural         ER_R !
Erode urban         ER_U !
Kancheepuram rural  KC_R !
Kancheepuram urban  KC_U !
Kanyakumari rural   KK_R !
Kanyakumari urban   KK_U !
Karur rural         KRR_R!
Karur urban         KRR_U!
Kodambakkam chen nonKBM_N!
Kodambakkam chen sluKBM_S!
Krishnagiri rural   KR_R !
Krishnagiri urban   KR_U !
Madhavaram chen non MVM_N!
Madhavaram chen slumMVM_S!
Madurai rural       MA_R !
Madurai urban       MA_U !
Manali chen non slumMNI_N!
Manali chen slum    MNI_S!
Nagapattinam rural  NG_R !
Nagapattinam urban  NG_U !
Namakkal rural      NM_R !
Namakkal urban      NM_U !
Nilgiris rural      NI_R !
Nilgiris urban      NI_U !
Perambalur rural    PE_R !
Perambalur urban    PE_U !
Perungudi chen non sPGI_N!
Perungudi chen slum PGI_S!
Pudukottai rural    PU_R !
Pudukottai urban    PU_U !
Ramanathapuram ruralRA_R !
Ramanathapuram urbanRA_U !
Royapuram chen non sRPM_N!
Royapuram chen slum RPM_S!
Salem rural         SA_R !
Salem urban         SA_U !
Sholinganallur chen SNR_S!
```

11. SITE.REC

```
Sholinganallur chen SNR_N!
Sivaganga rural     SI_R !
Sivaganga urban     SI_U !
Teynampet chen non sTMT_N!
Teynampet chen slum TMT_S!
Thanjavur rural     TJ_R !
Thanjavur urban     TJ_U !
Theni rural         TH_R !
Theni urban         TH_U !
Thoothukudi rural   TK_R !
Thoothukudi urban   TK_U !
Tiruchirapalli ruralTC_R !
Tiruchirapalli urbanTC_U !
Tirunelveli rural   TI_R !
Tirunelveli urban   TI_U !
Tiruppur rural      TP_R !
Tiruppur urban      TP_U !
Tiruvallur rural    TL_R !
Tiruvallur urban    TL_U !
Tiruvannamalai ruralTV_R !
Tiruvannamalai urbanTV_U !
Tiruvarur rural     TR_R !
Tiruvarur urban     TR_U !
Tiru-vi-ka nagar cheTVK_N!
Tiru-vi-ka nagar cheTVK_S!
Tiruvottiyur chen noTVT_N!
Tiruvottiyur chen slTVT_S!
Tondiarpet chen non TDT_N!
Tondiarpet chen slumTDT_S!
Valasaravakkam chen VVM_N!
Valasaravakkam chen VVM_S!
Vellore rural       VE_R !
Vellore urban       VE_U !
Villupuram rural    VL_R !
Villupuram urban    VL_U !
Virudhunagar rural  VR_R !
Virudhunagar urban  VR_U !
```
